# Eye Movements during Measurements of Visual Vertical in the Poststroke Subacute Phase

**DOI:** 10.1523/ENEURO.0279-24.2024

**Published:** 2025-01-16

**Authors:** Yasuaki Arima, Kae Nakamura, Kimihiko Mori, Shingo Hashimoto, Masanori Wakida, Hironori Ishii, Kimitaka Hase

**Affiliations:** ^1^Department of Rehabilitation Medicine, Kansai Medical University Hospital, Hirakata, Osaka 573-1191, Japan; ^2^Department of Physiology, Kansai Medical University, Hirakata, Osaka 573-1010, Japan; ^3^Faculty of Rehabilitation, Kansai Medical University, Hirakata, Osaka 573-1136, Japan; ^4^Department of Rehabilitation Medicine, Kansai Medical University, Hirakata, Osaka 573-1010, Japan

**Keywords:** disengagement, eye movements, stroke, subjective visual vertical, unilateral spatial neglect

## Abstract

The subjective visual vertical (VV), the visually estimated direction of gravity, is essential for assessing vestibular function and visuospatial cognition. In this study, we aimed to investigate the mechanisms underlying altered VV perception in stroke participants with unilateral spatial neglect (USN), specifically by examining their eye movement patterns during VV judgment tasks. Participants with USN demonstrated limited eye movement scanning along a rotating bar, often fixating on prominent ends, such as the top or bottom. This suggests a reflexive response to visually salient areas, potentially interfering with accurate VV perception. In contrast, participants without USN showed broader scanning around the center of the bar. Notably, participants with USN without frontal lobe lesions occasionally exhibited extended scanning that included the bar’s center, which was associated with accurate VV judgments. These findings suggest that (1) a tendency to fixate on peripheral, prominent areas and (2) frontal lobe involvement in disengaging and redirecting spatial attention may influence VV perception in USN. Based on these results, targeted rehabilitation strategies that encourage individuals with USN to extend their visual scanning beyond prominent endpoints and include central areas could improve VV accuracy. This study highlights the specific eye movement behaviors contributing to VV misperception, emphasizing the importance of training that broadens scanning to improve VV perception effectively.

## Significance Statement

While subjective visual vertical (VV) assessment requires visually judging a rotating bar's verticality, stroke participants with unilateral spatial neglect (USN) exhibit impaired VV perception. However, the underlying mechanisms remain unclear. Through analyses of eye movement patterns, this study demonstrates shorter eye-scan lengths along the bar and focused gaze on conspicuous parts of the bar among participants with USN, which disrupts the integration of vestibular and visuospatial processes. Furthermore, USN participants without frontal lobe lesions show occasional improvements in VV perception with broad eye scans around the center of the bar, indicating a frontal lobe’s role in visuospatial disengagement and updating the mechanisms in verticality sensation. These findings underscore the significance of understanding visual search patterns for effective USN rehabilitation.

## Introduction

Individuals with stroke often develop lateropulsion (Nolan et al., 2022), a postural disorder in which they actively lean away from the unaffected side toward the affected side. Lateropulsion has been viewed as an attempt to align the orientation of body to a tilted, distorted representation of verticality relative to gravity. Verticality perception disorders typically manifest in stroke patients with unilateral spatial neglect (USN; Dai et al., 2021; Embrechts et al., 2023; Lafitte et al., 2023). USN, often associated with lesions of the right brain hemisphere, is a neuropsychological disorder characterized by impaired perception, orientation, and response to stimuli contralateral to the lesion side (i.e., often left; Heilman et al., 1993; Vallar, 1998; Mesulam, 1999; Corbetta et al., 2005). Remarkably, verticality perception disorders in lateropulsion have been considered a form of spatial neglect in three dimensions (Dai et al., 2021).

Verticality perception relies on the integration of the vestibular, somatosensory, and visual sensations (Brandt et al., 1994; Pérennou et al., 2008; Barra et al., 2010; Karnath, 2015). Among them, the internal model based on visual information has been estimated through subjective visual vertical (VV; Brandt et al., 1994; Bonan et al., 2006; Pérennou et al., 2008; Barra et al., 2010; Yang et al., 2014; Piscicelli and Perennou, 2017). VV is typically assessed as the deviation between the actual and perceived straight vertical orientation. Stroke participants with USN exhibit a significantly greater contralesional deviation of VV, accompanied by higher VV variability (Kerkhoff and Zoelch, 1998; Yelnik et al., 2002; Saj et al., 2005; Bonan et al., 2006; Fukata et al., 2020; Mori et al., 2021). However, few studies have investigated the nature and underlying mechanisms of altered verticality perception in relation to specific visuospatial cognition.

One way of obtaining insights into visuospatial perception during VV measurement in individuals with USN is by analyzing eye movement patterns, as visuospatial analyses are linked to eye movements (Leek et al., 2012), and the direction of gaze is tightly coupled to attention orientation (Hoffman and Subramaniam, 1995). Thus far, numerous studies have reported altered exploratory eye movements in individuals with USN while they searched for relevant targets in a static visual scene (Behrmann et al., 2001; Karnath, 2015). Gaze distribution is generally skewed toward the non-neglected right space, and scan areas are reduced in both at-rest and in-search tasks in individuals with USN (Vuilleumier et al., 2007; Berger et al., 2008; Ptak et al., 2010; Machner et al., 2012; Karnath, 2015; Takamura et al., 2016; Delazer et al., 2018; Walle et al., 2019).

This study had two primary objectives. First, we aimed to characterize the eye-scan paths of individuals with and without USN while they performed a task involving the judgment of the verticality of a bar. Notably, we used a rotating bar as a dynamic visual stimulus rather than a static one; VV has been assessed using visual stimuli via a limited dynamic component where participants adjusted the orientation of the bar or examiners presented the bar at a randomly changing angle (Piscicelli and Perennou, 2017). However, in real-life scenarios, one must process dynamically changing sensory information while voluntarily controlling posture based on accurate visuospatial perception. We hypothesized that individuals with different visuospatial perceptions, specifically those with and without USN, would exhibit characteristic visuospatial analyses as quantified based on their eye movement patterns. Second, we sought to explore whether a trial-by-trial relationship existed between the eye-scan paths and verticality judgments in participants with USN. Understanding how specific eye-scan paths influence verticality judgments could inform novel rehabilitation strategies for individuals with USN.

## Materials and Methods

### Participants

This study included 42 participants who had experienced their first episode of hemorrhagic or ischemic hemispheric stroke within the previous 40 d. Participants with brainstem, cerebellar, or subarachnoid hemorrhagic stroke, brain tumors, bilateral brain lesions, a history of previous stroke, or any other neuropsychiatric disorders and those who had difficulty understanding instructions or communicating due to severe aphasia or cognitive impairment were excluded. Thirty healthy individuals (aged 43–79 years, mean 64.2) with no history of neuropsychiatric disorders were included as normal controls (NCs). All participants self-reported that they were right-handed. All participants provided informed consent before inclusion in the study, and the research protocol was approved by the Ethics Committee Kansai Medical University (reference number, 2015123). All the experiments were conducted in accordance with the principles of the Declaration of Helsinki.

### Neuropsychological assessment

To assess USN, the participants were screened based on three standard neuropsychological tests selected from the Japanese version of the Behavioral Inattention Test (Shinkoh Igaku Shuppan)—the line bisection, star cancellation, and flower copying tasks. In the line bisection task, participants were asked to mark the center of three 8 in horizontal lines. Each line was scored based on the degree of deviation from the true center, with a maximum of three points per line. The cutoff score was seven out of nine points or lower (Schenkenberg et al., 1980). In the star cancellation task, 56 small stars were randomly distributed on the panel, interspersed with 52 large stars, 10 short words, and 13 letters. After two small stars were crossed by an experimenter, as an instruction, the participants were asked to cross out the remaining 54 small stars. The cutoff score was 51 points or less, i.e., overlooking more than three stars. Participants copied a flower with petals, leaves, and a stem in the flower copying task. The omission of at least one feature of a figure is scored 0, indicating USN (Gainotti and Tiacci, 1971). Participants were classified as having USN [USN(+)] if they tested positive on at least one of the assessments. All evaluations were conducted on the same day.

Among the 42 participants with stroke included in this study, 17 were classified as USN(+). All USN(+) participants had right hemispheric damage (RHD). The remaining 25 participants were categorized as without USN [USN(−)]; of them, 10 had RHD, and 15 had left hemispheric damage (LHD). A previous study included 11 USN(+), 5 USN(−)RHD, 12 USN(−)LHD, and 30 NC participants (the citation is omitted for double-blind peer review).

### VV measurement

Each participant was positioned upright with their head and trunk secured using a headrest, belts, and cushions solidly but also comfortably. Thus, although deviations in head and body posture rarely occurred, the participant’s head and body positions were visually monitored and corrected, if necessary, to maintain accuracy of postural and eye position (see below, Measurement of eye movements). This standardization of the postural setting is crucial for comparing participants with different postural balance abilities (Piscicelli and Perennou, 2017).

**Figure 1.**
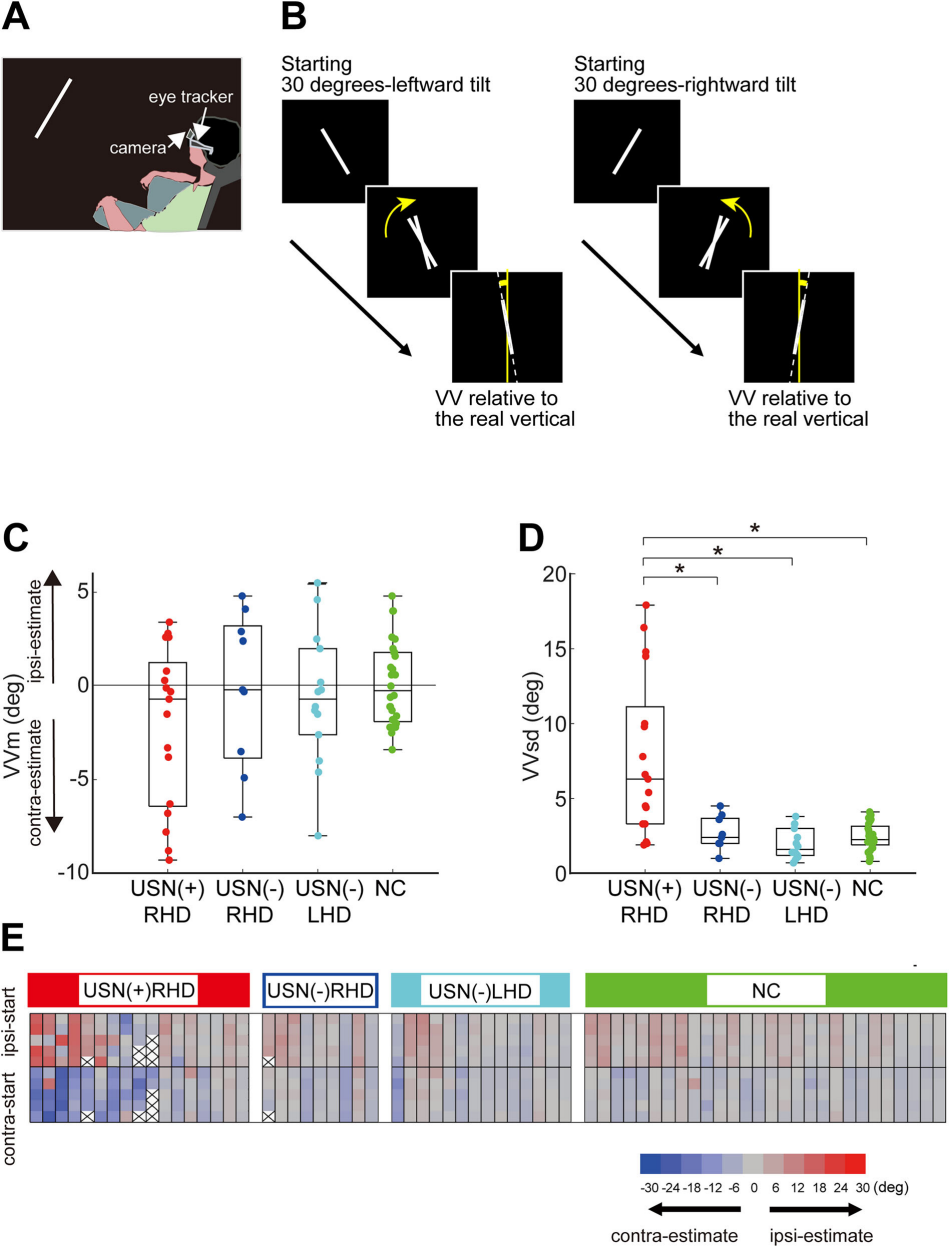
Measurement procedure of the subjective VV and eye movement. ***A***, Experimental setup. ***B***, A luminous bar, initially tilted 30° to the left or right, was gradually rotated toward the vertical around its center on a dark background until the participant judged that it was precisely vertical. The VV was defined as the discrepancy (in degrees) between the subjectively judged and true vertical orientation (0°). ***C***, ***D***, Orientation (***C***) and variability (***D***) of the VV in different groups. The box plot center mark indicates the median; a box’s top and bottom edges indicate the 25th and 75th percentiles, respectively. Each dot indicates the data for one participant. USN(+)RHD, participants with USN and right hemisphere damage (RHD) (*n* = 17); USN(−)RHD, participants without USN and with RHD (*n* = 9); USN(−)LHD, participants without USN and with LHD (*n* = 14); NC, normal control (*n* = 28). ***C***, VVm, the mean (degree) of VV in all trials for each participant, as the VV orientation. Negative values indicate deviation of the VV toward the contralesional direction; positive values indicate deviation of the VV toward the ipsilesional direction. ***D***, VVsd, the standard deviation of VV in all trials for each participant, as the intraindividual variability in VV. Asterisks indicate significance levels with Bonferroni’s correction (adjusted *p* < 0.05/6, Wilcoxon rank-sum test). ***E***, Trial-based VV values for each participant. Participants are arranged on the horizontal axis in a descending order of VVsd. The VV is shown for five trials starting from an ipsilesional tilt (top half) and five trials from a contralesional tilt (bottom half). Red and blue indicate deviation from the vertical to the ipsilesional and contralesional sides, respectively. The color density indicates the extent of deviation in degrees (see the color scale below the panel). Cross marks indicate missing values. Additional statistical analyses, including correlations between VVm, VVsd, and the severity of USN, are shown in Extended Data Figure 1-1.

The experiment was conducted in a dark room in which a luminous line, called a “bar,” measuring 30 cm in length (corresponding to a visual angle of 17.1°) and 1 cm in width (visual angle of 0.6°), was projected onto a screen positioned 1 m away from the participants using a projector located behind and above the participant. To avoid the influence of visual reference cues from the edges of the projected rectangular area, an oval-shaped cutout was placed in front of the projector lens (Fig. 1*A*; Mori et al., 2021).

10.1523/ENEURO.0279-24.2024.f1-1 Figure 1-1 Correlation between VVm, VVsd, and the severity of USN assessed using neuropsychological tests. Each black circle indicates the mean value of each USN(+)RHD participant. Overlayed plots represent participants with lesions in distinct brain areas—five green circles with frontal cortex lesions, four red asterisks with frontal eye field lesions, a pink plus with supplementary eye field lesions, and two inverse blue triangles with parietal eye field lesions. It should be noted that some of these plots include overlapping participants. ρ and p indicate the result of Spearman’s rank correlation. In the line bisection test, 9 was the best score; in the star cancellation test, 54 was the best score; in the flower copying task, 0 indicates the omission of at least one feature. Download Figure 1-1, TIF file.

The bar was initially tilted 30° to the left or right from the vertical orientation and rotated at a speed of 1° every 1.2 s in the clockwise or counterclockwise direction, respectively (Fig. 1*B*). Participants were instructed to inform the operator verbally when they perceived the bar as vertical. At that point, the operator would stop the rotation of the bar. Each VV value was recorded in 1° increments. Each participant completed 10 trials, with 5 trials each starting from the left-tilted and right-tilted positions. The order of the trials was randomized. During the intertrial interval, participants were instructed to close their eyes to minimize any additional visual reference. VV was assessed by quantifying the degree of deviation in the angle of the bar from the true vertical orientation. Negative values indicated tilts toward the contralesional side (leftward/counterclockwise in NCs), whereas positive values indicated tilts toward the ipsilesional side (rightward/clockwise in NCs) of VV. In the previous study, participants were allowed to verbally instruct the operator to make fine adjustments after the initial judgment (the citation is omitted for double-blind peer review), and these statically adjusted VV data were then analyzed. In the present study, however, we analyzed initially judged VV to examine the direct relationship between eye movements prior to VV judgment and dynamic verticality perception.

### VV data analyses

For each participant, we computed the mean VV (VVm) across 10 trials to represent the overall orientation of VV and the standard deviation of VV (VVsd) as a measure of the intraindividual variability of VV. To compare the VVm and VVsd between the USN(+)RHD, USN(−)RHD, USN(−)LHD, and NC groups, the Kruskal–Wallis test (*p* < 0.05) and the Wilcoxon signed-rank test with Bonferroni’s correction (*p* < 0.05/6) as a post hoc test were performed. The one-sample Wilcoxon signed-rank test was used to compare whether the VVm in each group was significantly different from the true vertical orientation (0°).

### Measurement of eye movements

We recorded the eye position and bar orientation on the screen using a noninvasive video-based glasses–type eye tracker (iView ETG; SensoMotoric Instruments). The eye tracker allowed us to measure the eye position across a range of up to 80° horizontally and 60° vertically. The sampling rate for recording eye position was set at 30 Hz, with an accuracy of 0.5°. The eyeglasses used in the eye tracker were equipped with a camera that captured the image of the rotating bar on the screen at a resolution of 1,280 × 960 pixels, meaning that each degree of the visual angle corresponded to ∼16 pixels on the screen. At the start of VV measurement, calibration with at least three points was performed to ensure accurate tracking. Furthermore, during the 10 trials, the position of the participant’s eyes on the monitor and the position of the head and trunk (as described above, VV measurement) were visually monitored. Recalibration was performed to maintain accuracy whenever the head and trunk positions were corrected or the eye position was confirmed to drift.

### Analysis of the eye-scan path

The video captured by the eye tracker was converted into a sequence of 30 JPEG-formatted images per second, matching the sampling rate. From each of these images, we extracted the position of the rotating bar and the eye using functions available in the MATLAB Image Processing Toolbox—“hough,” “houghpeaks,” and “houghlines” (bar) and “Imfindcircles” (eye position). Images in which the eye position was outside the measurement range (horizontal, 80°; vertical, 60°) were excluded from further analyses.

**Figure 2.**
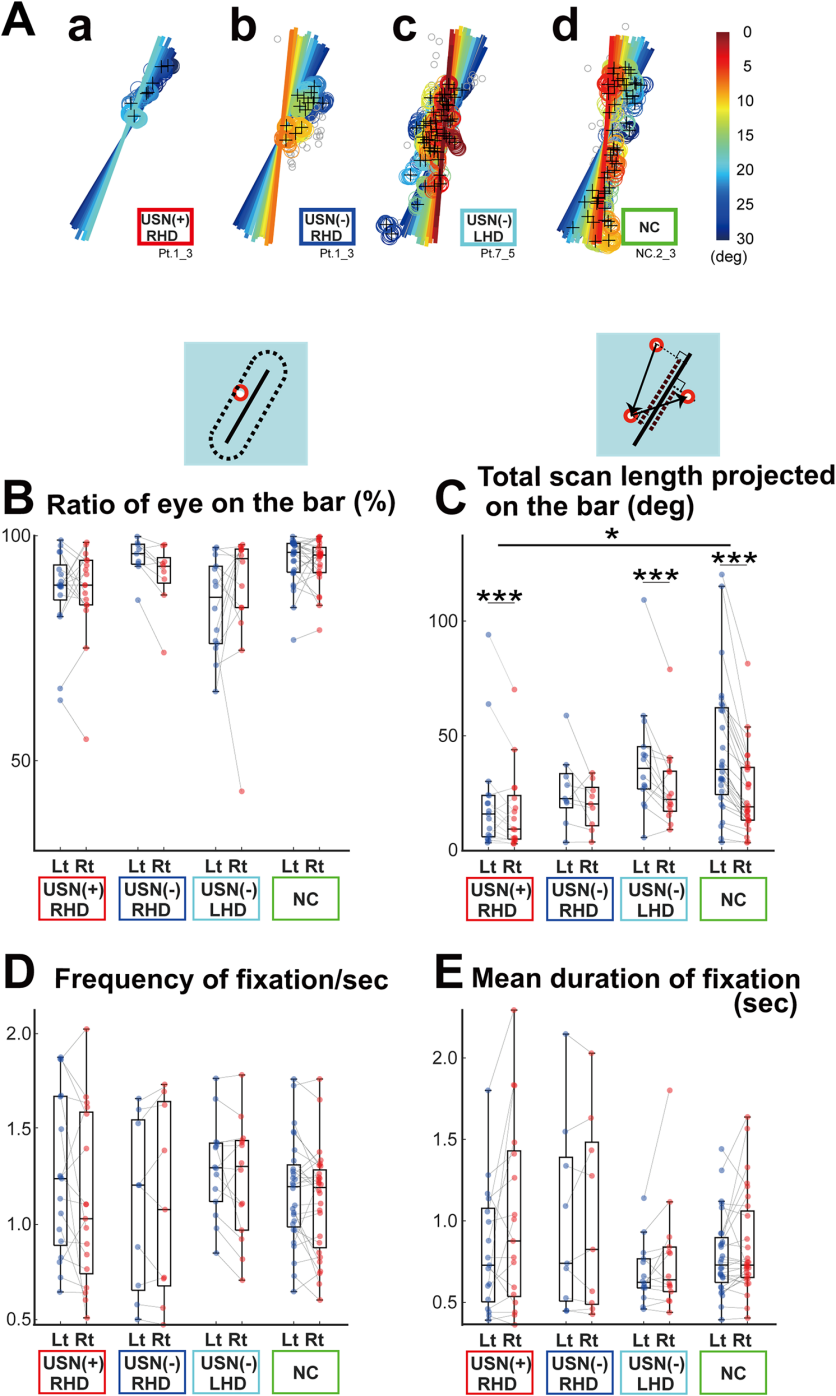
Each participant group showed characteristic eye-scan paths. ***A***, Examples of eye position and bar orientation. The panels illustrate one initial right-tilt trial from a 30° tilt until the participant responded that the bar was vertical. ***a***, USN(+)RHD, participants with USN; ***b***, USN(−)RHD, participants without USN with RHD; ***c***, USN(−)LHD, participants without USN with LHD; ***d***, NC, normal controls. Colored circles and the black + indicate the eye position detected as a “fixation” (see Materials and Methods), and gray circles indicate the eye positions that failed to meet the criteria of a fixation. The orientation of the bar and the corresponding fixation circles for each moment are in the same color: 30° tilt, blue, and red, straight. ***B-E***, Quantitative characterization of eye-scan paths during the subjective VV measurement. ***B***, “Ratio of an eye on the bar,” the ratio of the duration for which the eye position (red circle) is within a 2° distance from the bar relative to the positions over the whole trial duration (see an inset above the graph). ***C***, “Total scan length projected on the bar,” the total sum of eye movement length projected on the bar (dotted lines in an inset above the graph). ***D***, Frequency of fixation (for the definition of “fixation”; see Materials and Methods). ***E***, Mean duration of fixation. For each panel, asterisks indicate the main effect of group difference as assessed using a GLMM and Tukey’s test (*p < 0.05; ***p < 0.001). The trials were categorized based on their initial tilt direction: Lt (leftward or counterclockwise tilt, blue) and Rt (rightward or clockwise tilt, red), and each participant's mean values for both directions are connected by a line. Additional statistical analyses, including correlations between VV measures and the eye movement measures in USN(+)RHD participants, are shown in Extended Data Figure 2-1.

We analyzed the eye position data starting at the presentation of the bar until the participant responded. We computed the following parameters to characterize eye-scan paths relative to the rotating bar. The “ratio of the eye position on the bar” represents the duration for which the eye position was within 2° of the distance from the bar relative to the total duration of a trial (Fig. 2*B*). A ratio close to 1 indicated that participants faithfully kept looking at a part of the rotating bar. Second, the “total scan length projected on the bar” was computed as the total sum of the eye movement length projected on the bar (Fig. 2*C*). As the bar constantly changed its orientation, this parameter reflected the amount of visuospatial exploration through eye movements, along with the direction of the bar. Third, the “frequency of fixation” and “mean duration of fixation” were computed as described previously (Krassanakis et al., 2014). Briefly, fixation identification was based on two spatial parameters and one minimum duration. First, beginning with the initial image, the mean values of the horizontal and vertical coordinates of the eye position were computed until the distance between the mean point up to that point and the current record exceeded 1°. Once the distance surpassed 1°, a new fixation cluster was formed. Second, the distance between the mean point and every record within the cluster was calculated for each cluster. If the distance of a record exceeded the predefined tolerance value (0.9°), that particular record was excluded from the computation of fixation coordinates. The fixation coordinates were then computed as the mean point of each cluster, with the duration equal to the difference in the passing times between the last and first records of the cluster. Third, after applying the two spatial constraints described above, fixation clusters with a duration shorter than 150 ms were excluded. The “frequency of fixation” and “mean duration of fixation” for each trial were then computed.

10.1523/ENEURO.0279-24.2024.f2-1 Figure 2-1 Correlation between VVm, VVsd, and the eye movement measures in USN(+)RHD participants. The format follows that of Fig. 1-1, using the same symbols to represent lesions in specific brain regions for consistency. Download Figure 2-1, TIF file.

Trial duration was defined as the time interval from when the bar began to rotate until the participant verbally instructed the operator to stop upon perceiving it to be straight. Note that the trial duration differed depending on VV [e.g., USN(+)RHD group participants often responded earlier that the rotating bar was vertical; Fig. 1*E*]. Thus, “total scan length reflected on the bar” and “number of fixations” were normalized by the duration of each trial (i.e., divided by total trial duration).

### Statistical analyses of eye-scan path

A generalized linear mixed model (GLMM) was used to analyze the eye-scan path data, accounting for the correlated nature of repeated measures for the same participant while allowing for missing observations*.* The data were log-transformed for analysis. The fixed effects included the group (group), the number of trials within a session (trial), the initial orientation of the bar (initial orientation), and the interaction between the group and initial orientation. Estimated means and standard errors were calculated for each parameter using the model. These estimates were compared between groups using Tukey’s method (*p* < 0.05). Statistical analyses were performed using the R software (version 4.2.2). All detailed results of the statistical analyses are available in Extended Data Statistical Table. The R script for the analysis of the “total scan length projected on the bar” was as follows:

model1 <- glmer(Total scan length projected on the bar ∼

Group + Trial + Initial orientation +

Group: Initial orientation +

(1|subject ID), data = df, family = Gamma(link = “log”))

10.1523/ENEURO.0279-24.2024.t1-1 Statistical Table. Download Statistical Table, DOCX file.

### Analysis of the distribution of eye positions

**Figure 3.**
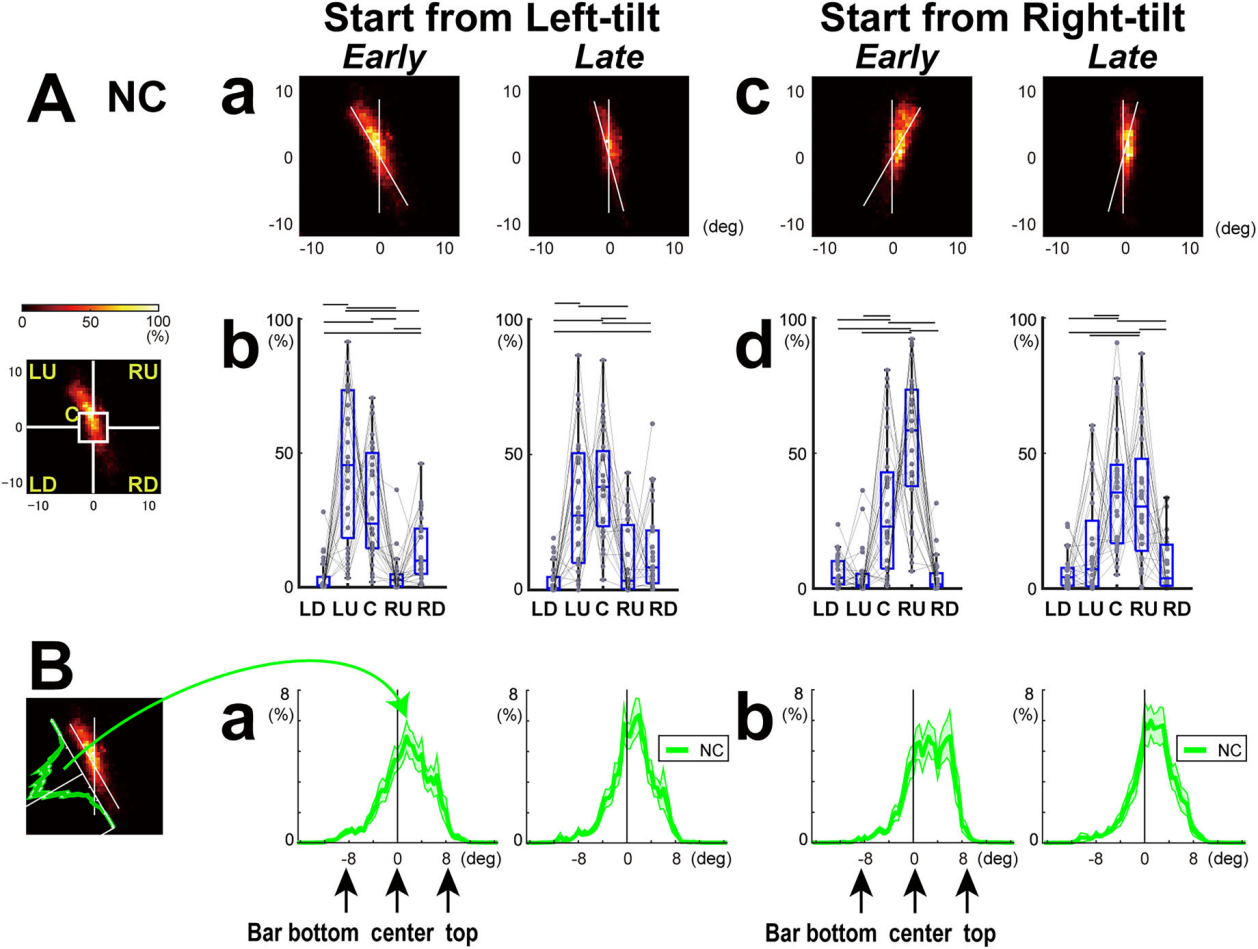
The distributions of eye positions during the measurement of subjective VV for normal control participants (NC). ***a***, ***c***, Mean of the ratio of eye position density two-dimensional maps for NC participants, separately for the initial direction of the bar, start from “left-tilt” or “right-tilt” and during the “early” (30° to 15° tilt) and “late” (15° tilt and more) phases of a trial. *X* and *Y* axes indicate degrees from the center of the rotating bar. White lines indicate the real vertical and 30° (early) or 15° (late) tilts. ***b***, ***d***, Relative frequencies of eye positions in five different visual spaces. ***C***, A central square of 4 × 4°; LU, left-up; LD, left-down; RU, right-up, and RD, right-down represent spaces excluding the central square. In each box plot, the center mark indicates the median, and the top and bottom edges of the box indicate the 25th and 75th percentiles, respectively. Data of each participant are connected by lines. The lines above the box plot signify significant differences between spaces. ***B***. The distribution of eye position relative to the bar. The eye position on the top half of the bar was defined as positive. The data signify the mean and SE of the eye position of all NC participants.

We also analyzed the distribution of eye positions during the measurement of VV using the following procedure. Initially, eye positions in the visual space from each image frame were recorded and combined to create a relative frequency map constructed as a two-dimensional histogram using bins of 0.5°. This process was performed separately for each participant, considering two conditions: (1) trials with initial rightward or leftward tilt and (2) the earlier phase (30–15° tilt) and later phase (15° tilt and onward) of each trial. The resulting fixation density histograms were then visualized as heat maps (Fig. 3*Aa*). To characterize eye positions for each group, we computed the mean of the relative frequency maps for each participant.

For a quantitative comparison of the eye position distribution, we calculated the relative frequencies of eye positions in two spatial map measures for each participant under different conditions (i.e., initial tilt direction and earlier/later phase within a trial). The first was the eye positions in five two-dimensional visual spaces, including a central square of 4 × 4° as well as the left-up (LU), left-down (LD), right-up (RU), and right-down (RD) spaces outside the central square (excluding the central 4 × 4°; Fig. 3*Ab*,*d*). The Kruskal–Wallis test was conducted to examine the differences in eye position frequency between these spaces, followed by pairwise comparisons using Dunn’s test (*p* < 0.05). The second measure was the eye position projected on the rotating bar (Fig. 3*Ba*,*b*). The mean relative frequency of eye position on the bar was calculated for each participant and condition. The difference between the participant groups was assessed using ROC analyses with bootstrapping (1,000 times) for each bar position for every 0.5° bin.

### Analyses of the effect of longer eye scans on VV

To assess the effect of the total eye-scan length on VV for each USN(+)RHD participant, we computed the “VV improvement index” as [the absolute value of VV for a trial with the shortest eye-scan length projected on the bar]− [the absolute value of VV for a trial with the longest eye-scan length projected on the bar], separately for the initial orientation of the bar (Fig. 6*A*). Positive values indicated more improvement in VV with longer eye scan. If the average of the “VV improvement index” for ipsilateral- and contralateral-start trials was positive, we classified the participant as having “VV improved with longer eye scans”; if the average “VV improvement index” was negative, the participants were classified as having “VV not improved with longer eye scans.”

### Brain mapping and lesion analysis

Each participant underwent standard clinical radiological assessments, including brain magnetic resonance imaging [MRI; diffusion-weighted imaging (DWI), T2-weighted, or fluid-attenuated inversion recovery] or computed tomography (CT), according to standard protocols for individuals with stroke at our university. All lesion maps were reviewed by a physical therapist and a rehabilitation physician. The brain lesions in the eye movement-related cortical areas were identified by referring to published data (Luna et al., 1998)—frontal eye field (FEF; Lobel et al., 2001; Vernet et al., 2014), supplementary eye field (SEF; Grosbras et al., 1999), and parietal eye field (PEF; Muri et al., 1996). Fisher’s exact test was used to compare the relationship between areas of brain lesions involved in eye movements and VV improvement.

The lesion map of subacute ischemic lesions was manually outlined on the DWI, and the volume of the lesion was calculated as the number of voxels using the MRIcron software (Rorden et al., 2007; available online at https://people.cas.sc.edu/rorden/mricron/index.html). Subsequently, tissue-type segmentation with partial volume estimation was used to calculate the total normalized brain volume (including separate estimates of gray matter and white matter) using a combination of MRIcron and Statistical Parametric Mapping 12 (http://www.fil.ion.ucl.ac.uk/spm; Wellcome Department of Cognitive Neurology) in MATLAB (MathWorks). The calculated volume of the lesion was normalized by total brain volume. The Wilcoxon rank-sum test was used to compare the lesion size between the groups with and without VV improvement.

### Software accessibility

The code for data analyses may be made available upon reasonable request to the corresponding authors.

## Results

### Participant profiles and lesion anatomy

One USN(−)RHD, one USN(−)LHD, and two NC participants were excluded from the analyses due to unstable eye movement data. Thus, data of 17 USN(+)RHD, 9 USN(−)RHD, 14 USN(−)LHD, and 28 NC participants were analyzed. Table 1 summarizes the data regarding the age, sex, and time since stroke occurrence in each group, and Table 2 provides a detailed profile of all the included participants.

**Table 1.** Characteristics of study participants

	USN(+)RHD	USN(−)RHD	USN(−)LHD	NC
Number of participants	17	9	14	28
Age (years)	64.4 ± 10.9	69.1 ± 6.9	60.9 ± 11.9	63.8 ± 9.9
Sex (F/M)	3/14	8/1	12/2	11/17
Days since stroke	21.3 ± 10.0	7.0 ± 2.5	14.8 ± 3.8	
VVm (deg)	−0.7 [−6.4, 1.3]	−0.2 [−3.9, 3.2]	−0.7 [−2.6, 2.0]	−0.3 [−1.9, 1.8]
VVsd (deg)	6.3 [3.3, 11.1]	2.4 [2.0, 3.7]	1.6 [1.2, 3.0]	2.3 [1.9, 3.1]

VVm, mean of visual vertical; VVsd, standard deviation of visual vertical; USN, unilateral spatial neglect; USN(+)RHD, USN with right hemisphere damage; USN(−)RHD, no USN, with right hemisphere damage; USN(−)LHD, no USN, with left hemisphere damage; NC, normal control

Data are presented as either the mean ± standard deviation (for age and time since stroke) or as median (25th, 75th percentile; for VVm and VVsd).

**Table 2.** Demographic and clinical data of participants

Group	Participants	Lesion side	Lesion location	Age years)	Sex (F/M)	Days since stroke (days)	Line bisection	Star cancellation	Copying	VVm	VVsd
USN(+)RHD	Pt. 1	R	SLF/ Ic/ S/ C/ Ins	69	F	16	0	17	0	−0.3	17.9
	Pt. 2	R	Par/ T/ TPJ	59	M	8	8	52	0	0.8	9.8
	Pt. 3	R	SLF/ Ic/ S/ Th	63	M	4	9	49	1	−0.1	1.9
	Pt. 4	R	SLF/ Par/ Ins	64	M	5	9	48	1	3.4	2.0
	Pt. 5	R	SLF/ PF/ M1	72	M	5	9	53	0	−3.3	3.3
	Pt. 6	R	SLF / C/ O	53	F	24	5	22	0	−0.7	2.1
	Pt. 7	R	SLF/ C/ O	76	M	14	1	41	0	2.8	4.5
	Pt. 8	R	SLF/ PF/ M1/ T/ Ins/ FEF	64	M	9	7	41	0	−1.5	10.0
	Pt. 9	R	SLF/ Ic/ S/ C/ Ins/ Th	77	M	27	9	37	1	2.6	3.3
	Pt. 10	R	SLF/ PF/ FEF	75	F	10	7	22	0	2.6	14.5
	Pt. 11	R	SLF/ Ic/ S/ C/ Ins	56	M	25	9	44	0	−3.8	4.4
	Pt. 12	R	SLF/ Th/ Ic/ C	57	M	23	6	53	1	−6.3	5.4
	Pt. 13	R	SLF/ Par/ T/ TPJ/ O/ V1/ IPL/ SPL/ PEF	73	M	6	0	14	0	0.3	16.4
	Pt. 14	R	SLF/ S/ Ins/ C/ Th	56	M	30	9	47	0	−9.3	6.3
	Pt. 15	R	SLF/ S/ Ins/ Par/ PF/ M1/ FEF	73	M	40	7	46	1	−7.8	7.8
	Pt. 16	R	SLF/ S/ Th/ SPL	34	M	11	9	27	0	−6.8	6.6
	Pt. 17	R	SLF/ S/ Par/ T/ TPJ/ O/ PF/ IPL/ SPL/ FEF/ SEF/ PEF	74	M	32	4	12	0	−8.8	14.8
USN(-)RHD	Pt. 1	R	Ic/ C	69	M	4	9	54	1	4.1	3.9
	Pt. 2	R	SLF/ Ins	72	M	7	9	54	1	−7.0	2.0
	Pt. 3	R	S/ C	75	F	8	9	53	1	2.9	3.6
	Pt. 4	R	S/ C	72	M	5	9	48	1	−4.9	2.6
	Pt. 5	R	S1/ Par	75	M	11	9	54	1	−0.2	2.0
	Pt. 6	R	Th	72	M	7	9	53	1	−0.3	2.4
	Pt. 7	R	SLF/ Th/ Ic/ S/ C/ Ins	66	M	5	9	54	1	2.4	4.5
	Pt. 8	R	SLF/ Ic/ S/ C	70	M	9	9	53	1	4.8	2.0
	Pt. 9	R	SLF/ C	51	M	5	8	54	1	−3.5	1.0
USN(-)LHD	Pt. 1	L	Th	60	M	5	9	52	1	2.0	1.1
	Pt. 2	L	C	65	M	6	9	54	1	4.6	3.3
	Pt. 3	L	S1	60	M	7	9	54	1	2.5	3.0
	Pt. 4	L	SLF/ C	66	M	14	9	54	1	−4.0	1.2
	Pt. 5	L	C	66	M	11	9	53	1	−1.5	1.4
	Pt. 6	L	SLF/ C/ S1/ Par/ IPL/ SPL	52	F	14	9	54	1	−4.6	2.0
	Pt. 7	L	SLF/ C/ PF/ SPL	58	M	8	9	54	1	−0.3	0.9
	Pt. 8	L	S/ C	65	M	7	9	54	1	5.5	3.3
	Pt. 9	L	Th/ Ic/ S/ Ins	48	M	22	9	54	1	−1.1	0.7
	Pt. 10	L	Th/ Ic	68	M	7	9	54	1	−8.0	3.8
	Pt. 11	L	Ic/ C/ Th	73	M	17	9	54	1	−2.6	1.3
	Pt. 12	L	SLF/ Ic/ S/ C	76	F	18	9	54	1	−0.2	2.4
	Pt. 13	L	PF/ S1/ M1	68	M	8	9	54	1	−1.3	1.4
	Pt. 14	L	SLF/ S/ C	27	M	10	9	54	1	0.2	1.8

L, left; R, right; SLF, superior longitudinal fasciculus; Ic, internal capsule; S, striatum; C, corona radiata; Ins, insula; Par, parietal cortex; T, temporal cortex; TPJ, temporoparietal junction; O, occipital cortex; PF, prefrontal cortex; M1, primary motor cortex; S1, primary sensory cortex; Th, thalamus; V1, primary visual cortex; IPL, inferior parietal lobule; SPL, superior parietal lobule; FEF, frontal eye field; SEF, supplementary eye field; PEF, parietal eye field; VVm, mean of visual vertical; VVsd, standard deviation of visual vertical; USN, unilateral spatial neglect; USN(+)RHD, USN with right hemisphere damage; USN(−)RHD, no USN, with right hemisphere damage; USN(−)LHD, no USN, with left hemisphere damage.

All 17 USN(+)RHD participants had lesions in the right hemisphere. Among them, 16 (94%) had lesions in the superior longitudinal fasciculus (SLF), with 7 (41%) in the insula and 9 (53%) in the superior parietal lobule, 3 in the inferior parietal lobule, 7 (41%) in the striatum, 4 in the thalamus, and 3 in the temporal cortex or temporoparietal junction. Five participants had lesions in the prefrontal cortex. Other affected areas included the internal capsule, corona radiata, primary motor cortex, primary sensory cortex, and occipital cortex. Thus, the USN(+)RHD participants had lesions in representative neglect-associated brain areas (Karnath et al., 2004).

### VV assessment

Figure 1*C* depicts the VVm data. Of the 17 USN(+)RHD participants, 11 had negative VVm values, indicating an overall deviation to the contralesional side, although it did not reach statistical significance (one-sample Wilcoxon signed-rank test, median [25th, 75th percentile] = −0.7 [−6.4, 1.3], *p* = 0.107). Significant deviations were not observed in the USN(−)RHD (−0.2 [−3.9, 3.2]), USN(−)LHD (−0.7 [−2.6, 2.0]), and NC (−0.3 [−1.9, 1.8]) groups. Moreover, VVm did not differ significantly among the groups (Kruskal–Wallis; *χ*^2^ = 1.936; *p* = 0.585; df = 3).

In contrast, VVsd differed significantly between the groups (Fig. 1*D*; Kruskal–Wallis; *χ*^2^ = 22.006; *p* < 0.001; df = 3). Post hoc analysis revealed that the VVsd in the USN(+)RHD group (6.3 [3.3, 11.1]) was significantly higher than those in the other groups (USN(−)RHD, 2.4 [2.0, 3.7]; USN(−)LHD, 1.6 [1.2, 3.0]; and NC, 2.3 [1.9, 3.1]).

Among the USN(+)RHD participants, VVsd increased with the increased severity of USN, as assessed using neuropsychological test scores. A significant correlation was observed between VVsd and line bisection test (Spearman’s rank correlation; *ρ* = −0.64; *p* = 0.007) and star cancellation test (*ρ* = −0.54; *p* = 0.031). While the correlation with flower copying test scores was not significant, a trend was observed (*ρ* = −0.45; *p* = 0.078). VVm did not show a significant correlation with the severity of USN (Extended Data Fig. 1-1). Details of the statistical comparisons, including post hoc tests, are provided in Extended Data Statistical Table.

As reported in the previous study (Mori et al., 2021), the large variability in VV (VVsd) was primarily because USN(+)RHD participants responded before the rotating bar straightened or too early. Specifically, they responded when the bar was still leftward-tilted for initially leftward-tilt trials and still rightward-tilted for initially rightward-tilt trials (Fig. 1*E*).

### Characteristic eye-scan paths in participants with USN during verticality judgment

During VV measurement, participants searched around the rotating bar using eye movements. Figure 2*A* shows example trials wherein the bar was initially tilted 30° to the right (shown in blue) and then rotated gradually in the counterclockwise direction (shown in yellow, then red) until the participant responded upon perceiving the bar to be vertical. The eye positions of USN(+)RHD participants (Fig. 2*Aa*) were restricted to the upper half of the bar, whereas those of USN(−)RHD (Fig. 2*Ab*) and USN(−)LHD (Fig. 2*Ac*) participants were distributed more widely, around the bar’s center. The eye positions of NC participants (Fig. 2*Ad*) were widely distributed from the upper to the lower portion of the bar. Notably, all participants, including NCs, responded before the bar straightened (in this case, when the bar was still tilted to the right), consistent with previously reported findings (the citation is omitted for double-blind peer review; Fig. 1*E*).

We computed several measures for a further quantitative analysis of the eye-scan path. The mean of the “ratio of the eye on the bar” (Fig. 2*B*) was over 0.8 for most participants in all groups, indicating that their eye position was consistently close to or on the rotating bar. Although the GLMM showed a significant effect on the “group” in the eye on the bar ratio (Fig. 2*B*; group effect; *χ*^2^ = 8.49; df = 3; *p* = 0.036), a post hoc analysis did not reveal a significant between-group differences.

While the eye positions of all participants tended to be on the bar, scan paths differed significantly between the groups. Analysis of the “total scan length projected on the bar” (Fig. 2*C*), a measure of how much the participant visually deviated with the orientation of the bar, revealed significant variations among the groups (Fig. 2*C*; *χ*^2^ = 11.08; df = 3; *p* = 0.011). Moreover, the post hoc analysis revealed that this value was significantly lower in the USN(+)RHD group than in the NC group (*p* = 0.028) for leftward-tilt trials. Our analyses also revealed significant variations depending on the initial orientation of the bar (*χ*^2^ = 109.33; df = 3; *p* < 0.001) and an interaction between the group and initial orientation of the bar (*χ*^2^ = 14.26; df = 3; *p* = 0.003). The post hoc analysis revealed that the “total scan length projected on the bar” was significantly higher for initial left-tilt trials than for initial right-tilt trials for USN(+)RHD, USN(−)LHD, and NC participants (*p* < 0.001), but it did not reach a significant different level for USN(−)RHD participants (*p* = 0.060).

Parameters related to fixation, including the number of fixations (*χ*^2^ = 2.29; df = 3; *p* = 0.51) and fixation duration (*χ*^2^ = 1.38, degrees of freedom = 3, *p* = 0.71), did not show consistent group effects (Fig. 2*D*–*E*).

Further analysis of the USN(+)RHD participants revealed no consistent correlation between VVm and the eye movement measures, whereas a significant negative correlation was observed between VVsd and the total scan length projected on the bar (Spearman’s rank correlation; *ρ* = −0.55; *p* = 0.031), indicating that longer eye scans were associated with more stable verticality perception (Extended Data Fig. 2-1).

### Distribution of eye position densities differed between the groups

**Figure 4.**
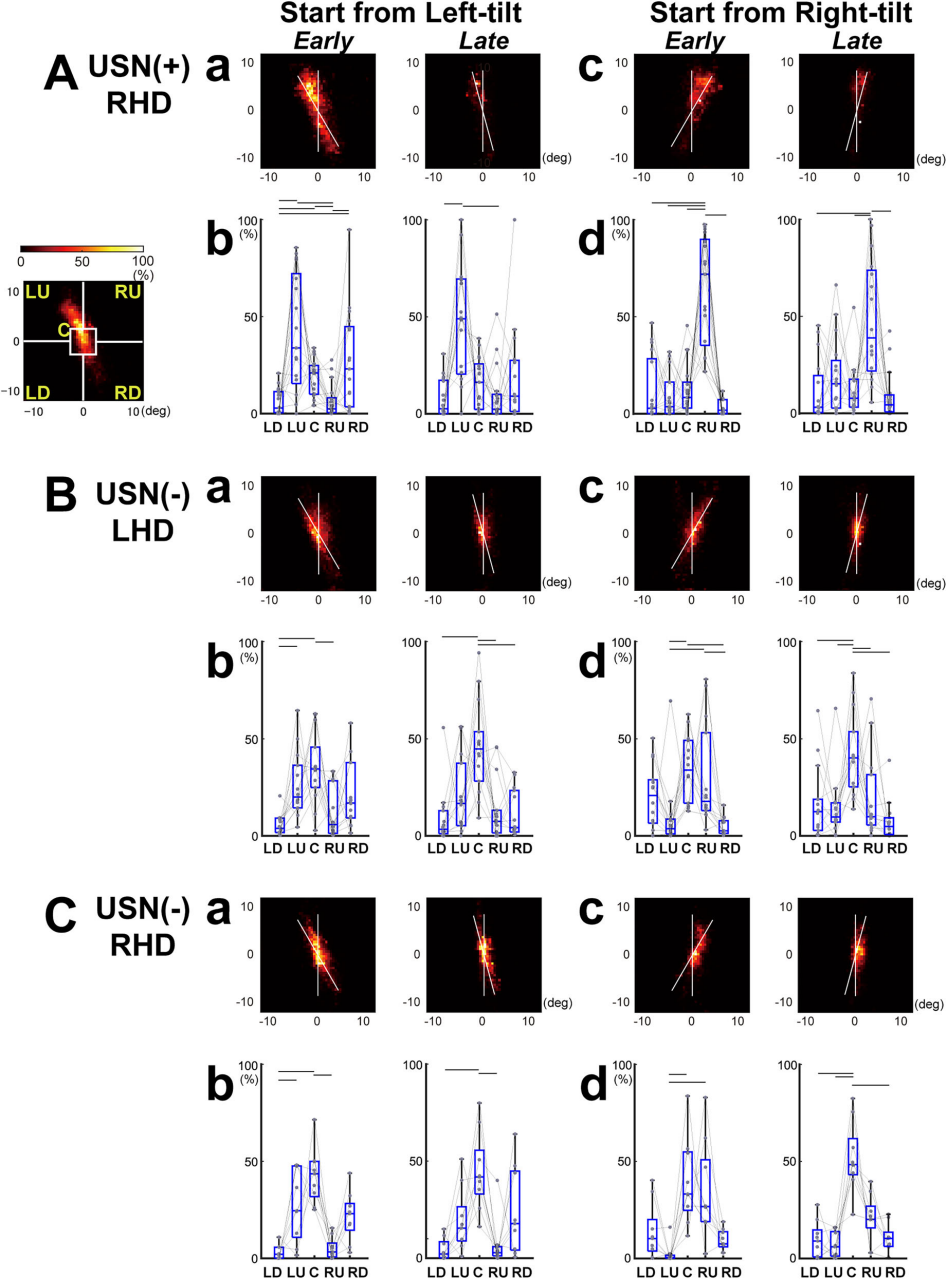
The different distributions of eye positions during the measurement of subjective VV between the groups. ***A***, USN(+)RHD: participants with USN; ***B***, ***C***, USN(−): participants without USN; RHD, right hemisphere damage; LHD, left hemisphere damage. Same format as Figure 3. For USN(+)RHD, the significant peak of the ratio of eye position was observed around the top part of the bar (***Ab***, “LU”; ***Ad*** “RU”) or lower part of the bar for left-tilt trials (***Ab***, “RD”). For USN(−)RHD and LHD, the significant peak of the ratio of eye position was observed around the center (**B**, ***Cb***, ***Cd***, “***C***”).

**Figure 5.**
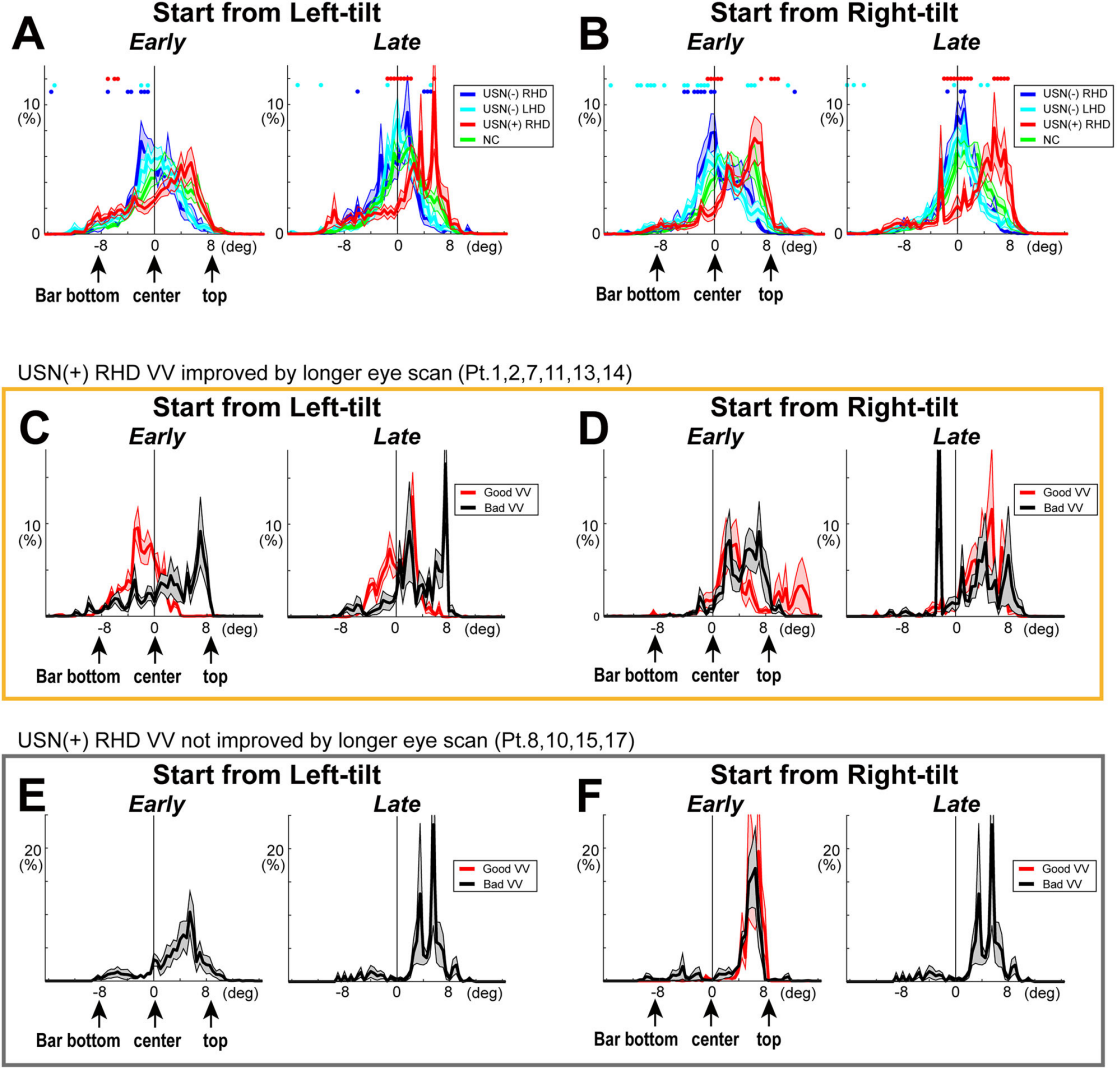
The distribution of eye position relative to the bar varied depending on the subject groups and subjective VV. The histograms signify the mean and standard error of the ratio of eye position frequencies reflected on the bar. The eye position on the top half of the bar was defined as positive. ***A***, ***B***, The distribution of eye position on the bar of USN(+)RHD in red, USN(−)RHD in blue, and USN(−)LHD in cyan are compared with NC in green (repeated presentation of Fig. 3*B*). Dots in each color indicate significant difference from NC (*p* < 0.05 ROC analyses using 1,000 times bootstrapping). ***C***, ***D***, The distribution of eye position on the bar of USN(+) RHD whose VV was improved by a longer eye scan on the bar (Fig. 6, USN(+) RHD Pts. 1, 2, 7, 11, 13, and 14). Red lines indicate trials with good VV (VV ≥ −4 or VV ≤ 4 (mean ± 2SD of VV data derived from NC, −4.3 < VV ≤ 4.5); black lines indicate trials with bad VV (VV ≤ −5 or VV ≥ 5). ***E***, ***F***, Same as ***C***, but of USN(+) RHD whose VV was not improved by a longer eye scan on the bar (Fig. 6, USN(+) RHD Pts. 8, 10, 15, and 17).

Studies have reported that rightward orientation bias is a sensitive measure of USN (Takamura et al., 2016; Delazer et al., 2018; Walle et al., 2019). To examine whether similar tendency was observed in the present study and whether the visuospatial bias or any other specific characteristics of sequences of eye position might affect verticality perception, we first created eye position density maps for each participant separately for the initial orientations of the bar, left- and right-tilt and during the early (15–30° tilt) and late (15° tilt and more) phases of a trial for each participant. We then pooled the data for all the participants in each group (Figs. 3*a*,*c*, 4*A*–*Ca*,*c*). For a quantitative comparison of the eye position distributions in two-dimensional spaces, we compared the relative frequencies of eye positions in five visual spaces, including the center (C) and the four quadrants—LU, LD, RU, and RD (Figs. 3*Ab*,*d*, 4*A*–*Cb*,*d*). We further performed quantitative estimates of the eye position frequencies relative to the bar, extending from the bottom, center, and top (Figs. 3*B*, 5).

In participants from the NC group, eye positions were primarily distributed from the center to the upper part of the bar (Fig. 3*Aa*,*c*). During both the early and late phases of bar rotation, eye positions of NCs tended to be LU and a central square (C) for trials starting from left-tilt (Fig. 3*Ab*) and toward RU and a central square (C) for trials starting from right-tilt (Fig. 3*Ad*). The eye position ratio on the bar was also high around the center, extending toward the upper part of the bar in the early phase (Fig. 3*Ba*,*b*, left columns), and the distribution tended to be narrowed in the late phase (Fig. 3*Ba*,*b*, right columns).

Compared with NC, USN(−)RHD and USN(−)LHD participants exhibited eye positions that were distributed more around the center of the bar during the early phase (Figs. 4*B*,*C*, 5*A*,*B*, left columns), with this central focus becoming more pronounced during the late phase (noted by highest concentration at “C” in Figs 4*B*,*C*, 5*A*,*B*, right columns).

USN(+)RHD participants showed characteristic eye position distributions. During the early phase, in the left-tilt trials, their eye positions were distributed around the top and bottom of the bar (Figs. 4*Aa*,*b*, left column; see high concentrations at “LU” and “RD”). In the right-tilt trials, eye positions were mainly distributed around the upper edge (Figs. 5*Ac*,*d*, left column; see high concentrations at “RU”). During the late phase, in both the left- and right-tilt trials, eye positions were restricted around the upper part of the bar (Figs. 4*Aa*–*d*, right columns; see high “LU” or “RU”). Thus, in leftward-tilt trials, USN(+)RHD participants’ eye positions are distributed around the upper part and less around the bar’s center or lower half. The eye position on the bar also confirmed a shifted distribution toward the bar top-end, especially prominent in the late phase (Figs. 5*A*,*B*, right columns) and lower part of the bar in the early left-tilt trials (Fig. 5*A*, left column).

### Longer eye-scan paths lead to improvement of VV in participants with USN

The aforementioned results showed that USN(+)RHD participants had large VV variability (Fig. 1*D*) and short “mean of total eye-scan path projected on the bar” (Fig. 2*C*). Such mean values represent the overall tendency for each participant; however, trial-by-trial variability in VV and eye-scan length raised the possibility that occasional long eye-scan length might compensate for verticality judgment even in USN(+)RHD participants.

**Figure 6.**
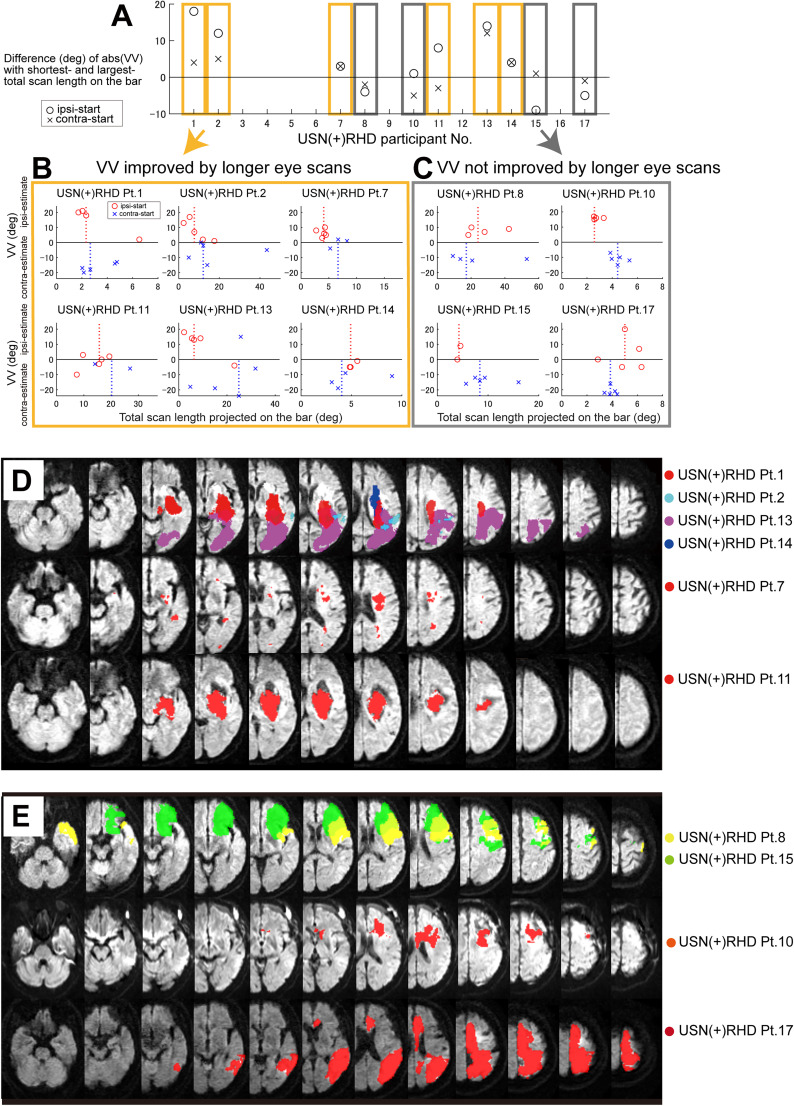
A subgroup of USN(+)RHD participants showed subjective VV improvement with longer eye-scan length on the bar. ***A***, For each USN(+)RHD participant, separately for initially left (contralateral)- and right (ipsilateral)-tilt trials, “VV improvement index”: [the absolute value of VV for a trial with the shortest eye-scan length projected on the bar] − [the absolute value of VV for a trial with the longest eye-scan length projected on the bar] was computed. Positive values indicate improvement of VV with longer eye scan. If the average of the indices for ipsilateral- and contralateral-start trials were positive (Pts. 1, 2, 7, 11, 13, and 14, colored in orange), we judged that the subject with “VV improved with longer eye scans”; if negative (Pts. 8, 10, 15, and 17, colored in purple), we assigned the subjects with “VV not improved with longer eye scans.” ***B***, ***C***, Trial-by-trial correlation between VV (vertical axis) and total scan length projected on the bar (horizontal axis) for USN(+)RHD participants. Trials that started from a rightward tilt are plotted using red open circles; those from a leftward tilt are plotted using blue cross marks; dotted vertical lines indicate the median value of the VV data computed separately for rightward- and leftward-tilt start trials. ***B***, A subgroup of “VV improved with longer eye scans” showed improvements in VV (i.e., close to zero on the vertical axis) with a longer total scan length projected on the bar (*X*-axis). ***C***, A subgroup of “VV not improved with longer eye scans” did not show improvement in VV even with a longer total scan length projected on the bar. ***D***, ***E***. Lesion anatomy derived using MRI and CT shows the extent of cortical and subcortical lesions in USN(+)RHD participants. ***D*** is of six participants, as in ***B***, who showed improvement; ***E*** is of four participants, as in ***C***, who did not show improvement of VV with longer eye scans. Data of participants excluded from comparisons based on trial selection criteria are shown in Extended Data Figure 6-1. Additional statistical analyses, including correlations between the normalized brain lesion size and VV measures or eye movement measures, as well as lesion locations in regions related to eye movement control, are shown in Extended Data Figure 6-2.

To ascertain whether longer eye-scan length was associated with lower VV deviation, we chose 10 USN(+)RHD participants with (1) “bad” VV trials, with VV values ≤−5 or ≥5 recorded in 1° increments (based on VV of NC; mean ± 2SD, −4.3 < VV ≤ 4.5); (2) a sufficient number of analyzed trials, i.e.,  >4; and (3) availability of good quality MRI scans [USN(+)RHD participants who failed to meet these criteria are reported in Extended Data Fig. 6-1]. We then computed the “VV improvement index” to determine the effect of longer eye scans on better VV (see Materials and Methods, Analyses of the effect of longer eye scans on VV) for each participant*.* We found that VV deviation was smaller as eye-scan path projected on the bar became longer in a subgroup of USN(+)RHD participants (Pts. 1, 2, 7, 11, 13, and 14; Fig. 6*A*,*C*, orange areas), whereas other USN(+)RHD participants (Pts. 8, 10, 15, and 17; Fig. 6*B*,*C*, gray areas) did not show such VV improvement. Figure 6, *B* and *C*, visualizes trial-by-trial effects of longer total scan length projected on the bar (*X*-axis) on each participant’s VV (*Y*-axis).

10.1523/ENEURO.0279-24.2024.f6-1 Figure 6-1 A. Trial-by-trial correlation between the subjective visual vertical (VV) (vertical axis) and total scan length projected on the bar (horizontal axis) in participants with unilateral spatial neglect (USN)(+) right hemispheric damage (RHD), which is not shown in Fig. 6 because (1) no “bad” VV trials (VV ≥ -4 or ≤ 4 (based on VV of NC, mean ± 2SD: -4.3 < VV ≤ 4.5)) occurred, (2) a sufficient number of analyzed trials, i.e., > 4 was not obtained, or (3) good quality MRI scans were not available. VVm and VVsd for the USN(+)RHD participants Pts. 3, 4, 5, 6, and 9 were within the normal range (mean ± 2SD of NC). The USN(+)RHD patient Pt. 12 had valid data from only one eye tracker trial. B. Lesion anatomy as assessed using MRI and CT showing the extent of cortical and subcortical lesions in the USN(+)RHD participants Pts. 3, 4, 5, 6, 9, and 12. The data for Pt. 16 were not available. Download Figure 6-1, TIF file.

10.1523/ENEURO.0279-24.2024.f6-2 Figure 6-2 Correlation between A. VVm, B. VVsd, C-F. Eye movement parameters and the normalized brain lesion size in USN(+)RHD participants. The format follows that of Fig. 1-1, using the same symbols to represent lesions in specific brain regions for consistency. Download Figure 6-2, TIF file.

**Figure 7.**
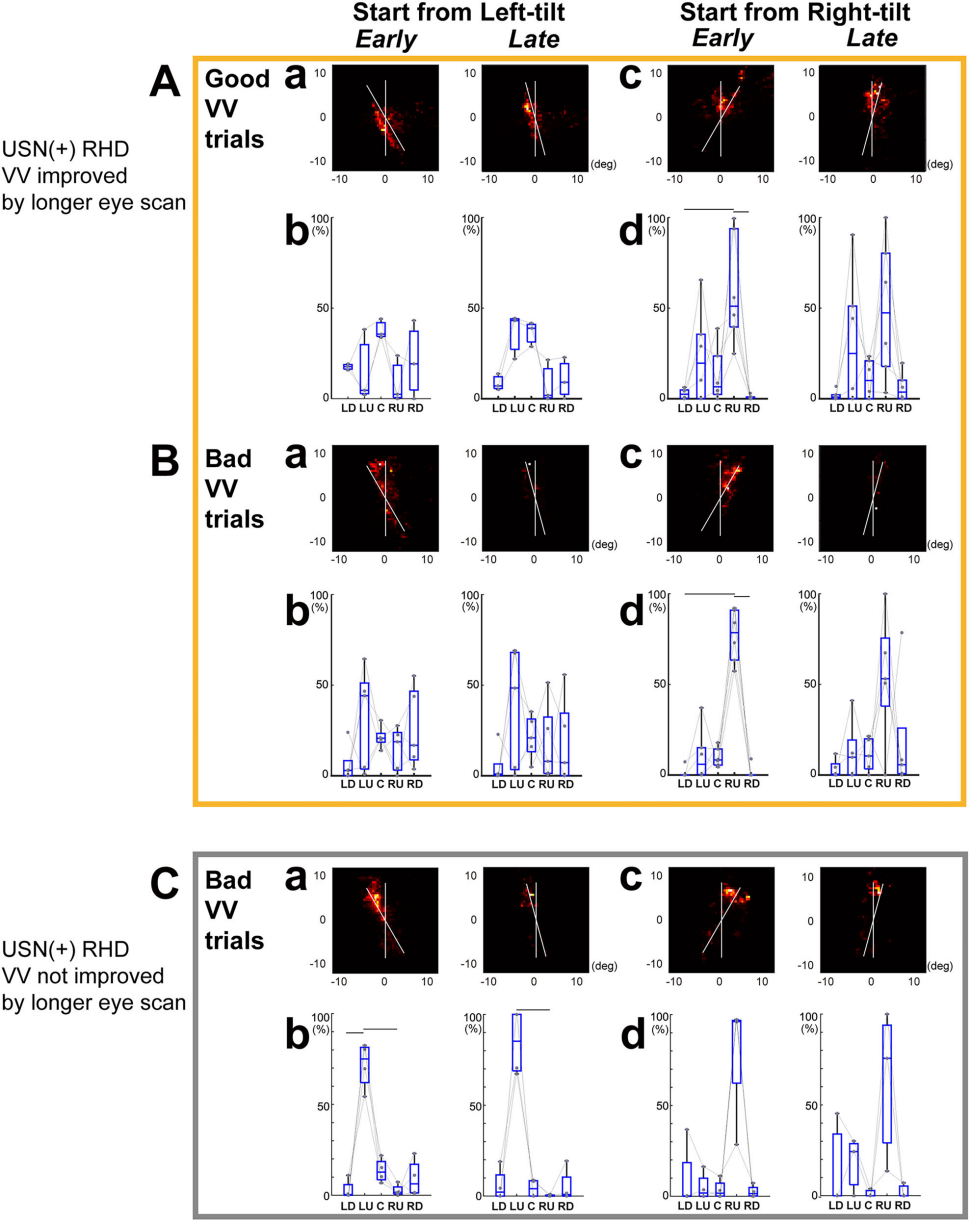
The distribution of eye scans was associated with subjective VV accuracy. ***A***, ***B***. The distributions of eye position for good VV (VV ≥ −4 or VV ≤ 4 (mean ± 2SD of VV data derived from NC, −4.3 < VV ≤ 4.5) and bad VV (VV ≤ −5 or VV ≥ 5) trials for USN(+)RHD participants (Pts. 1, 2, 7, 11, 13, and 14) whose VV was improved with longer eye scans along the bar. The two-dimensional mapping of the relative distribution of eye positions (***a***, ***c***) and relative frequencies of eye positions in five different visual spaces (***b***, ***d***) during good VV trials (***A***) and bad VV trials (***B***) are shown. Same format as Figure 4. ***C***, The distributions of eye position for bad VV trials for USN(+)RHD participants (Pts. 8,10, 15, and 17) whose VV was not improved with longer eye scans along the bar.

The eye position distributions also supported the correlation between eye-scan patterns and VV performance. As reported previously, the USN(+)RHD participant eye positions were generally concentrated at the top edge or lower part of the bar in left-tilt trials and at the top edge of the bar in right-tilt trials (Fig. 4*A*). To understand the relation between eye position distribution and VV performance, we split the trials into good VV trials (VV ≥ −4 or VV ≤ 4) and bad VV trials (VV ≤ −5 or VV ≥ 5) and compared the eye position distribution on the two-dimensional space (Fig. 7) and on the bar (Fig. 5*C*–*F*).

We found that in occasional good VV trials (−4 ≤ VV ≤ 4) observed in VV-improved participants 1, 2, 7, 11, 13, and 14, eye positions were deviated from the upper edge (Fig. 7*Aa*,*b*, high around “C”; Fig. 7*Ac*,*d*, high in both “RU” and “LU”). In bad VV trials (VV ≤ −5 or VV ≥ 5), the same participants’ eye positions remained around the upper part of the bar (Fig. 7*Ba*,*b*; highest for “LU”; Fig. 7*Bc*,*d*; highest for “RU”). In VV-unchanged participants 8, 10, 15, and 17 where bad VV trials always occurred, the eye positions were shifted to the upper part of the bar (Fig. 7*Ca*–*d*, high in “LU” or “RU”). The eye position plot relative to the bar also showed a clear tendency that bad VV trials were associated with a shift to the top edge of the bar (Fig. 5*C*–*F*, black lines).

### Lesion location and size affect verticality sensation but not eye movements

We examined the correlation between the normalized brain lesion size and verticality perception and eye movement measures (Extended Data Fig. 6-2). Although a significant positive correlation was observed between brain lesion size and VVsd (Spearman's rank correlation; *ρ* = 0.75; *p* = 0.001), no significant correlation was observed between the brain lesion size and VVm, as well as eye movement measures (Extended Data Fig. 6-2).

Additionally, we explored whether participants with lesions in the eye movement-related cortical areas expressed specific changes in eye movement measures for the present VV measure. Although the number of participants was limited, we did not observe systematic tendencies that specific lesions in the FEF, SEF, and/or PEF were correlated with specific changes in eye movement measures (Extended Data Fig. 6-2; *n* = 4 participants with FEF lesions; *n* = 1 SEF lesion; *n* = 2 PEF lesions).

Moreover, we observed that participants with or without VV improvement with long eye-scan paths had lesions in distinct brain areas. While participants whose VV improved in trials with a long eye-scan path had sparse lesions in the frontal areas (Fig. 6*D*; Table 2), those without VV improvement had lesions in the dorsolateral prefrontal cortical areas (Fig. 6*E*, top and middle rows; Table 2) or medial frontal cortical areas (Fig. 6*E*, lowest row; Table 2). With respect to these lesion locations associated with specific eye movements, all participants in the nonimproved VV group (*n* = 4) had lesions in the FEF, whereas none of the participants in the improved VV group (*n* = 6) had FEF lesions (*p* = 0.005, Fisher’s exact test). One participant in the nonimproved VV group had SEF lesions, whereas none in the improved group had SEF lesions (*p* = 0.40, Fisher’s exact test). Additionally, one participant in each group (nonimproved and improved VV) had a lesion in the PEF (*p* = 1.0, Fisher’s exact test). However, no significant difference in the lesion size was observed between the groups with without improved VV (*p* = 0.171, Wilcoxon rank-sum test).

## Discussion

### Eye movements differed in participants with USN during VV measurement

In the present study, the VV was quantified by asking the participants to respond when they perceived the rotating bar as being vertical. USN(+) participants exhibited highly variable VV, with a deviation of the mean in the contralesional direction, consistent with previous reports (Kerkhoff and Zoelch, 1998; Yelnik et al., 2002; Watanabe et al., 2020; Mori et al., 2021). The large difference in VVsd between the USN(+)RHD and other groups (Fig. 1*D*) and the significant correlation between neuropsychological test scores and VVsd (Extended Data Fig. 1-1) support the idea that VV misperception may even be considered a key feature of USN (Dai et al., 2021, 2022; Embrechts et al., 2023; Lafitte et al., 2023). However, few studies have investigated the mechanisms contributing to the association between USN and VV perception. As participants visually judged the rotation of the bar, we hypothesized that the altered VV observed in USN(+) participants would involve visuospatial perception supported by characteristic eye movements. The present study confirmed that eye movements during VV measurement of USN(+) participants differed from those of USN(−) participants.

First, while the eye positions of participants of all groups remained consistently on the bar during the experiment, as indicated by high ratio of eyes on the bar (Fig. 2*B*), USN(+)RHD participants showed a significant reduction in eye-scan length projected on the bar (Fig. 2*C*). This suggests that the eye positions of USN(+)RHD participants tended to “stick” to a particular position on the rotating bar, rather than searching along the bar. This is consistent with previous reports showing that USN(+)RHD participants exhibited a reduced scan area, manifested as a reduction in eye movement amplitudes, not only to the contralesional visual field but also to the ipsilesional visual field, during both at-rest and in-search tasks (Machner et al., 2012; Delazer et al., 2018).

The decrease in the length of the eye-scan path observed in USN(+)RHD participants may be associated with reduced exploratory eye movements due to impaired nonspatial and nonlateralized attention, alertness, and vigilance (Robertson et al., 1998; Niemeier and Karnath, 2000; Husain and Rorden, 2003; Malhotra et al., 2009). Alternatively, it may be explained by impaired attentional disengagement in participants with parietal injury (Posner et al., 1984), given that moving the eyes requires an engagement–disengagement process, as indicated by the eye position distribution described below.

Second, the eye position distribution differed among the groups. While NC and USN(−) participants’ eye positions were distributed around the center of the rotating bar, the USN(+)RHD participants showed a low ratio of looking at the center (Figs. 3, 4*B*,*C*, 5*A*,*B*). Instead, their eye positions are distributed around the bar’s upper edge (Figs. 4*A*, 5*A*,*B*). Eye movements in participants with USN tend to be reflexively elicited by low-level image statistics, such as visual saliency (Husain and Rorden, 2003), particularly at the periphery, but not during voluntary visual exploration (Machner et al., 2012). In the present study, the gaze might have been readily influenced by the tip of the bar, given its dynamic and conspicuous nature. This finding may also be consistent with a previous report of the robust disengagement deficit in participants with USN while following peripheral cues as opposed to central cues (Losier and Klein, 2001). Eye positions of USN(+)RHD participants were also distributed around the lower edge of the left-tilted bar in the right quadrant space but not in the lower left quadrant for right-tilt trials (Figs. 4*A*, 5*A*,*B*). This may reflect right visual hemifield dominance typically observed in participants with USN. The concentration of eye position in the peripheral part of the bar, as observed in the USN(+)RHD participants, may prevent the construction of an accurate allocentric reference frame, which may lead to altered VV. Conversely, scanning along the bar, particularly around the center, would lead to spatially balanced spatial coordinates.

In addition to the deviation of eye position being biased toward the edge of the bar or to the right hemifield, participants with USN have difficulty seeing, memorizing, and comparing the relative spatial position from the center of a rotating bar, attributable to impaired spatial working memory (Husain et al., 2001; Saj et al., 2020). Consequently, they may fail to update and construct the vertical representations of the dynamic visual stimulus spatially and temporally.

An unexpected result was that the participants, particularly NCs, showed longer total scan lengths projected on the bar for initially left-tilted trials than for initially right-tilt trials (Fig. 2*C*). A strong tendency to saccade to the left (Foulsham et al., 2013) or upward (Levine and McAnany, 2005) direction has been reported even in normal individuals. This left dominance has been explained by the fact that the right parietal lobe is disproportionally responsible for spatial attention (Foulsham et al., 2013), which declines in USN(+)RHD participants. Another notable finding in the NC group was the variability in visual scanning strategies during verticality judgment. While, overall, eye movements were longer among NCs (Fig. 2*C*), upon individual observations, the eye movement durations in some of them were as small as those of USN(+)RHD participants. NCs might employ various strategies to determine bar orientation, such as visuospatial analyses through eye movements and multisensory integration of somatosensory, vestibular, and visual sensations, even in the absence of eye movements. This approach, however, poses a challenge for participants with USN.

The present study also revealed that USN(−) participants’ VV perception performance did not show a significant difference from NCs. However, their eye movement data differed from those of NCs. Specifically, USN(−) participants, particularly the USN(−)RHD, exhibited relatively short eye-scan lengths, although the difference did not reach a significant level (Fig. 2*C*); this finding is consistent with a previous report (Ohmatsu et al., 2019). Moreover, USN(−) participants’ eye positions tended to be more concentrated around the center of the bar compared with those of NC and USN(+)RHD participants (Figs. 4*B,C*, 5*A*,*B*). In the USN(−) group, bar orientation may have been successfully ascertained using peripheral visual sensations with short eye movements while looking at the center. It has been reported that individuals without USN can discriminate the spatial location of the expansion focus using peripheral visual information in the optic flow direction task (Ogourtsova et al., 2020), indicating effective recruitment of peripheral vision to construct spatial information. Altogether, our results suggest that NC and USN(−) participants utilize at least two major strategies for perceiving the VV—updating and constructing the location of the rotating bar by either exploratory eye movements or peripheral visual perception without eye movements, supported by covert attention. Both of them may be integrated with somatosensory and vestibular sensations. NCs can utilize both strategies, whereas USN(−) participants may tend to rely on the latter strategy.

### Neural mechanisms underlying the role of eye movements in determining verticality

The present study demonstrated a relationship between spatial neglect, verticality perception, and eye movements. The changes in eye movements observed in stroke participants may simply be epiphenomena stemming from various degrees of damage to multiple brain regions associated with each function. Of the 17 USN(+)RHD participants, 16 had lesions in the SLF, linking the frontoparietal attention network with representational and perceptual neglect (Corbetta and Shulman, 2011). Seven had lesions in the insula, which is involved in visual–vestibular information processing (Brandt et al., 1994). Twelve had lesions in the FEF, SEF, and basal ganglia, all of which are involved in controlling eye movements.

The present study indicates the presence of a functional interaction between eye movements and verticality judgment of rotating visual stimuli in some USN(+)RHD participants; actively scanning the bar with sufficient length would help in estimating verticality accurately, even in participants with USN (Figs. 5*C*,*D*, 6).

How do eye movements along the bar contribute to better verticality judgment? In this study, the bar was constantly rotated in a predictable manner. To monitor the orientation of a rotating bar, it is necessary to consider its rotational speed (Keller and Johnsen, 1990) and actively search for its future location by sequentially shifting attention, which is often accompanied by eye movements. Visual information from one fixation to the next would then construct an accurate spatial coordinate. The integration of eye movements and verticality perception of a bar may be achieved by the spatiotemporal dynamic updating of multisensory integration. In particular, the predictive shift or spread of visual sensitivity “before” the generation of eye movements, called “predictive spatial remapping,” may contribute to better spatial analyses of sequentially moving objects, leading to accurate VV perception.

Neural mechanisms of such predictive remapping have been proposed. Animal studies have shown that the FEF, the cortical area that sends out the signal to the superior colliculus in the brainstem, the central mechanism of eye movement generation, also sends the corollary discharge to information about upcoming saccades (Rao et al., 2016).

Different oculomotor cortical regions have been proposed to serve different roles. The frontobasal ganglia–superior colliculus pathway is more involved in the volitional target selection and timing specification of cognitively demanding oculomotor tasks, whereas the parietal cortex is more involved in triggering reactive saccades and is important for determining the metrics of saccades (Terao et al., 2017). This functional difference supports that participants with frontal lesions, including in the FEF, show predominant impairments in a task involving voluntary spatial exploration but not when exploring natural scenes with reflexive orientation of attention (Heide and Kompf, 1998; Terao et al., 2016). The present study further emphasized the role of the frontal predictive remapping process for better VV perception. The findings indicated that lesion location, particularly in regions such as the FEF, plays a significant role in VV improvement. Participants with lesions in the FEF had more difficulty improving VV, highlighting the potential involvement of this region in supporting the visuospatial processing necessary for accurate verticality perception. In contrast, participants whose VV improved exhibited sparse lesions in the frontal cortical areas, suggesting that specific cortical areas may have unique contributions to VV-related eye movement adjustments. This result underscores the importance of considering lesion location, in addition to the lesion size, when assessing VV improvement potential. Interestingly, although the lesion size did not differ significantly between the groups, the presence of lesions in specific cortical areas, such as the FEF, was more strongly associated with impaired VV improvement. This suggests that lesion location may be more relevant than the lesion size in affecting visuospatial processing, particularly in terms of complex interactions between eye movement and verticality perception. This mechanism is critical for individuals with USN because, due to impaired integration of visual and somatosensory/vestibular information, their verticality perception may strongly depend on visual exploration.

### Clinical implications

The importance of accurate verticality perception for postural control (Bonan et al., 2006) highlights the need for systematic and regular assessment of VV in clinical practice as a complement to standard assessments of USN. The conventional assessments have not sufficiently captured the influence of dynamic visuospatial processes, particularly those mediated by eye movements. Our results suggest that a longer visual search, expanding along the bar and centering on the middle, integrated with vestibular and somatosensory information, would reduce the variability and improve the accuracy of verticality judgments. However, with impaired attention processes, especially in dealing with the prominent edges of the rotating bar, search behavior was significantly disrupted, resulting in distorted verticality perception.

The finding that an active, longer search of moving objects improves VV suggests new approaches to further enhance the accuracy of VV judgments for individuals with verticality perception deficits. For individuals without prefrontal lesions, encouraging visual scanning along the bar through verbal instructions may enhance VV perception accuracy because this approach can compensate for the decline of attentional processes. For those with prefrontal lesions, providing more salient visual prompts for attentional disengagement, such as external cues guiding attention toward the center of the bar and involving eye movements, may compensate for the reduced exploratory visual search (Corbetta and Shulman, 2011). Additionally, these prompts may be used to actively encourage broader visual exploration along the bar, ensuring effective engagement with the task.

Further study should address the contribution of different brain areas in verticality perception, in relation to eye movement and attention networks. In the present study, the number of participants, particularly those with lesions in the eye movement areas, was limited. It should also be noted that adding scandalized eye movement tasks such as visually and memory-guided saccade tasks (Terao et al., 2017) to the present behavioral task would reveal more fundamental mechanisms of the role of eye movements in verticality sensation.

In summary, analyses of eye movements during VV measurement using a constantly rotating bar revealed a characteristic pattern of altered visuospatial analysis that affects verticality judgment in participants with USN. These findings could provide a foundation for effective visuospatial rehabilitation for participants with postural disturbance.

### Data availability

The data supporting this study's findings are available on request from the corresponding author. The data are not publicly available due to privacy or ethical restrictions.

## Synthesis

Reviewing Editor: David Schoppik, New York University Grossman School of Medicine Decisions are customarily a result of the Reviewing Editor and the peer reviewers coming together and discussing their recommendations until a consensus is reached. When revisions are invited, a fact-based synthesis statement explaining their decision and outlining what is needed to prepare a revision will be listed below. The following reviewer(s) agreed to reveal their identity: Yasuo Terao.

Broad comments:

1. Regarding the association spatial neglect and verticality perception, and also wrong body orientation with respect to verticality please consider discussing ideas / framing in recent work by Lafitte et al. 2023 Annals of the NY Academy of Sciences, Embrechts et al 2023 Annals of PRM, Dai et al 2021 Neurology. We felt that these would help reformulate the hypothesis in the manuscript to take into account the fact that a wrong verticality perception (whatever the modality VV or PV) and its postural corollary lateropulsion are likely a form of spatial neglect bearing on gravity sensing.

2. The visual vertical is always subjective, please consider using the term "visual vertical (VV)."

3. The abstract could be more informative, with more explicit data-grounded statements.

4. Please consider avoiding the word "patient."

Title suggestion : Eye movements during measurements of visual vertical at the post-stroke subacute phase.

Introduction:

We were surprised that the manuscript authors start the introduction by the postural vertical and the link between postural disorders and a wrong postural vertical. This seemed to us to be out of the scope of this study/paper focused on the visual vertical. In the first sentence the manuscript reduces the postural imbalance to hemiplegia and wrong verticality perception which struck us as too simplistic and almost certainly incomplete. The references cited show that verticality perception is implied in the wrong body orientation with respect to gravity, one of the components of postural control and disorders (Dai et al Neurology 2021). In current parlance this postural behavior is called lateropulsion (see the recent consensus paper by Nolan et al) and the earlier terms, originally derived from "pusher syndrome" have been abandoned (see Dai et al). The paper by Karnath and al 2000 has never been replicated and data (only 5 patients) are highly suspected of a polarity error; we suggest deleting or at least qualifying this citation. The other component of postural control that can explain post-stroke postural disorders is postural stabilization, which suffers from deficits in motor commands and to somatosensation. We suggest reworking the first part of the Introduction to account for this literature.

Methods

1. How did the study control for lesion size when comparing eye movement patterns between patients with and without USN? See also the first comment under Results.

2. What methods were employed to ensure that participants maintained consistent head positioning during SVV measurements and to ensure the stability and accuracy of eye movement data during SVV assessments, especially given the potential for postural instability post-stroke?

3. (P5) How were the cutoff scores determined for the line bisection task, star cancellation task, and flower copying task and validated for the Japanese version of the Behavioral Inattention Test? Please explain a bit more about the cutoff beyond justifying the references.

4. (P7) When specifically did the recalibration of the eye tracker take place during the experiments?

5. How was the severity of USN in each patient assessed/scored?

Results

1. The manuscript reports that participants with or without SVV improvement with long eye-scan paths had lesions in distinct brain areas. However, the difference in the distribution of lesions is not quantitatively or statistically validated. We suggest registration to permit overlay of the lesions?

2. How did the researchers ensure that the observed differences in eye movements were specifically attributable to USN and not other confounding factors, such as direct damage to oculomotor brain regions?

3. In lines 388-389, the authors state "we split the trials into good SVV trials (SVV < 4.5 or SVV > -4.3) and bad SVV trials (SVV <= 4.5 or SVV > -4/.3)". According to the statement, the range of SVV for good and bad SVV trials are quite overlapping, except at SVV=4.5. Perhaps there is a mistake here

4. In figures 2 and 5, please provide explanations for the color of plots in a panel linked to each figure, to show which curve corresponds to which group of patients? There are captions to these figures but a panel may also help the reader.

5. In figure 5, there are also figures 5 and 5F. There are no legends given for these plots. Maybe the figures are wrongly labeled? Please clarify.

6. Regarding lesion analysis, were there any correlations between lesion size and the severity of SVV deviation or eye movement patterns? Also, were there any correlations between the severity of SVV deviation or severity of USN?

7. Was the variability in SVVsd among groups correlated with factors such as lesion size or specific locations within affected brain regions?

Discussion

1. In the discussion, the manuscript contrasts intentional spatial exploration versus natural scene exploration. Were there any specific criteria used to categorize eye movements during the SVV task as reflecting reflexive versus intentional processes? For this and other issues, please consider and refer to the following where appropriate. Terao Y, et. al. 10.2183/pjab.93.049.

2. How do the findings from this study contribute to our understanding of the prevalence and impact of USN and abnormal visual verticality in stroke rehabilitation, particularly in comparison to previous studies in the same field? Please discuss.

Conclusions

In the conclusions, the authors state that the findings could provide a foundation for effective visuospatial rehabilitation for participants with postural disturbance. What are the actual practical implications of your findings for developing targeted rehabilitation strategies aimed at improving SVV perception in stroke patients, especially those with USN? Considering the findings related to SVV improvement index and longer eye scan lengths, how might these insights inform personalized rehabilitation strategies for stroke patients with USN? Please discuss. While the paper mentions rehabilitation by inducing eye movements towards the neglected space, it doesn't clearly link this strategy back to the observed eye movement patterns (such as reduced scan lengths or peripheral fixation), leaving the reader to infer how these specific patterns relate to effective rehabilitation strategies. We feel the manuscript woudl be stronger if it elaborated a bit on these issues.

Possible typographical errors

Line 109: " participants s were categorized" - There is an extra "s" after "participants".

Line 450. Conversely, canning along the bar, →Conversely, scanning along the bar,

Line 515. brain stem→brainstem

Check the manuscript throughout for other possible typographical and grammatical errors
