# Supplementary figures and images for: Eye Movements during Measurements of Visual Vertical in the Poststroke Subacute Phase
Source: eNeuro. 2025 Jan 16;12(1):ENEURO.0279-24.2024. doi: 10.1523/ENEURO.0279-24.2024 (PMC11747974; doi:10.1523/ENEURO.0279-24.2024)

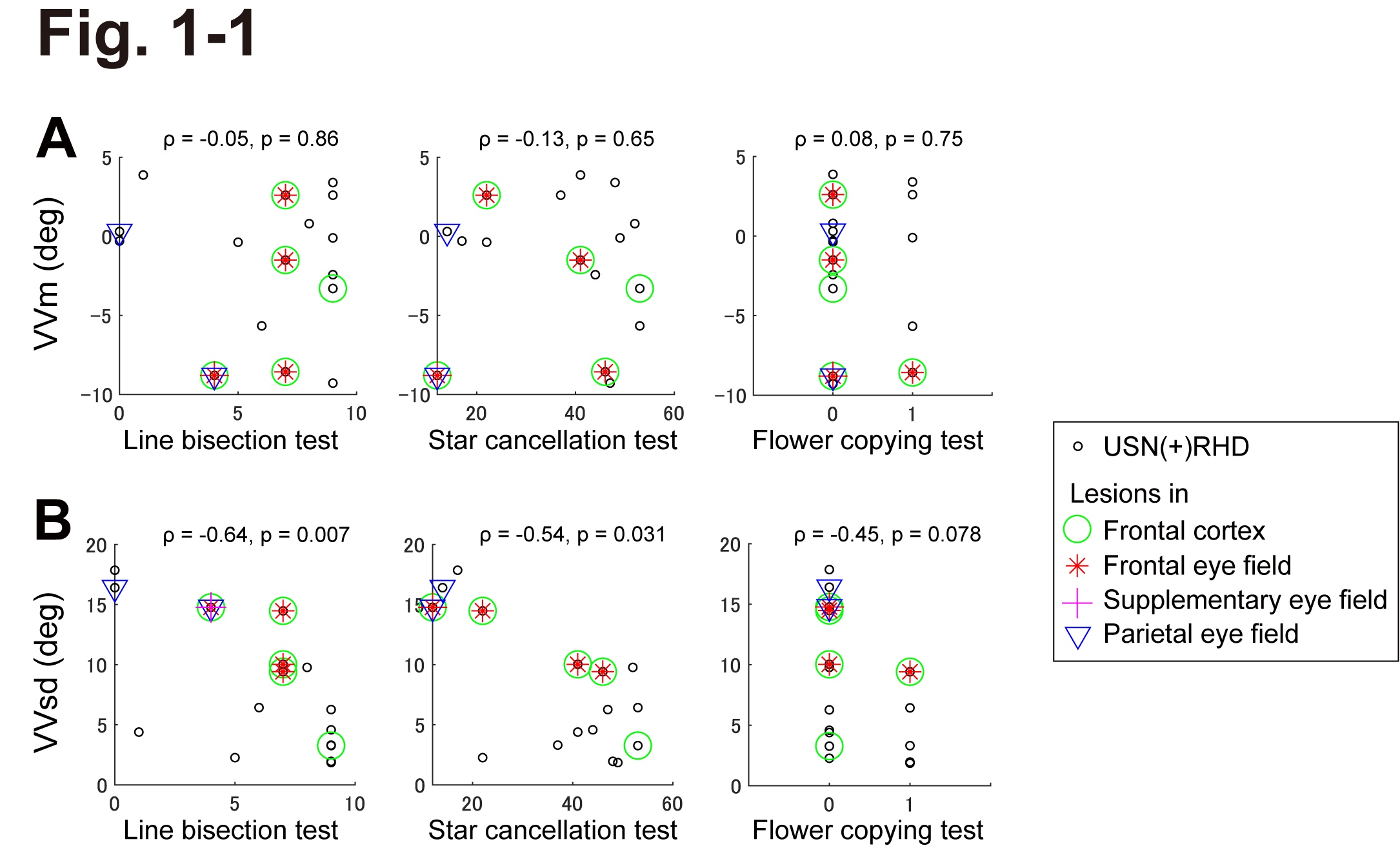

Supplement: Figure 1-1 — Correlation between VVm, VVsd, and the severity of USN assessed using neuropsychological tests. Each black circle indicates the mean value of each USN(+)RHD participant. Overlayed plots represent participants with lesions in distinct brain areas—five green circles with frontal cortex lesions, four red asterisks with frontal eye field lesions, a pink plus with supplementary eye field lesions, and two inverse blue triangles with parietal eye field lesions. It should be noted that some of these plots include overlapping participants. ρ and p indicate the result of Spearman’s rank correlation. In the line bisection test, 9 was the best score; in the star cancellation test, 54 was the best score; in the flower copying task, 0 indicates the omission of at least one feature. Download Figure 1-1, TIF file. [file eneuro-12-ENEURO.0279-24.2024-s001.tif]

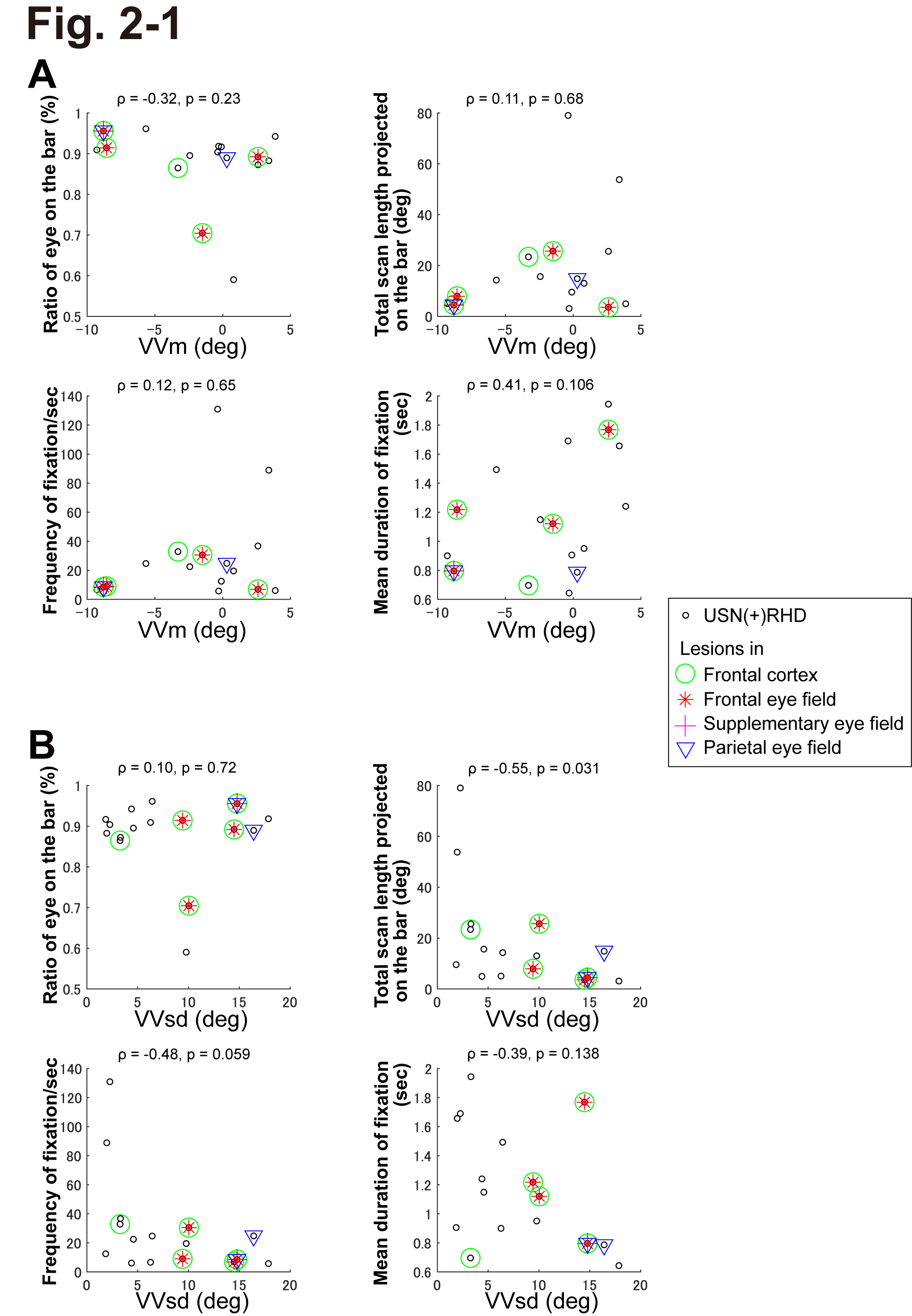

Supplement: Figure 2-1 — Correlation between VVm, VVsd, and the eye movement measures in USN(+)RHD participants. The format follows that of Fig. 1-1, using the same symbols to represent lesions in specific brain regions for consistency. Download Figure 2-1, TIF file. [file eneuro-12-ENEURO.0279-24.2024-s002.tif]

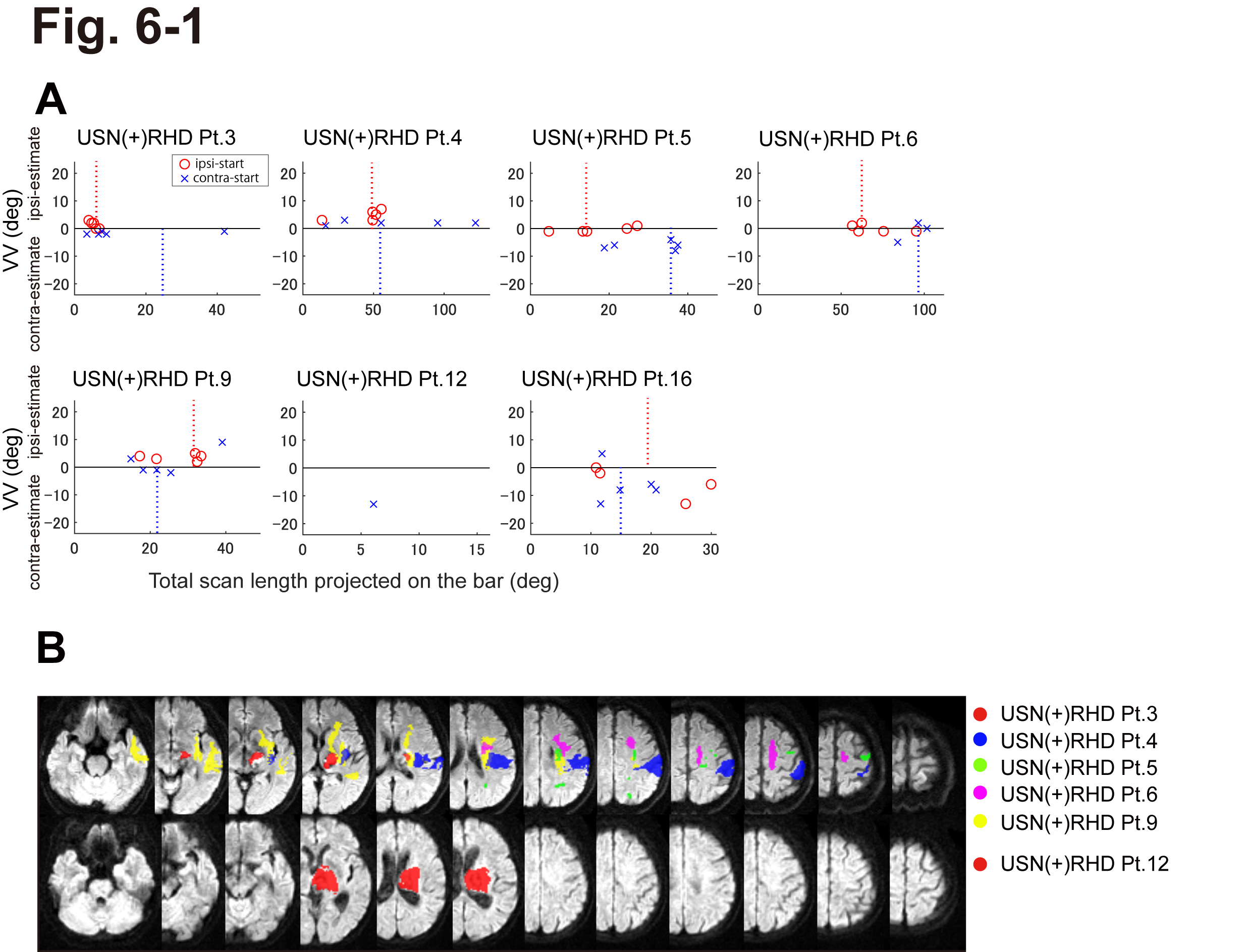

Supplement: Figure 6-1 — A. Trial-by-trial correlation between the subjective visual vertical (VV) (vertical axis) and total scan length projected on the bar (horizontal axis) in participants with unilateral spatial neglect (USN)(+) right hemispheric damage (RHD), which is not shown in Fig. 6 because (1) no “bad” VV trials (VV ≥ -4 or ≤ 4 (based on VV of NC, mean ± 2SD: -4.3 < VV ≤ 4.5)) occurred, (2) a sufficient number of analyzed trials, i.e., > 4 was not obtained, or (3) good quality MRI scans were not available. VVm and VVsd for the USN(+)RHD participants Pts. 3, 4, 5, 6, and 9 were within the normal range (mean ± 2SD of NC). The USN(+)RHD patient Pt. 12 had valid data from only one eye tracker trial. B. Lesion anatomy as assessed using MRI and CT showing the extent of cortical and subcortical lesions in the USN(+)RHD participants Pts. 3, 4, 5, 6, 9, and 12. The data for Pt. 16 were not available. Download Figure 6-1, TIF file. [file eneuro-12-ENEURO.0279-24.2024-s003.tif]

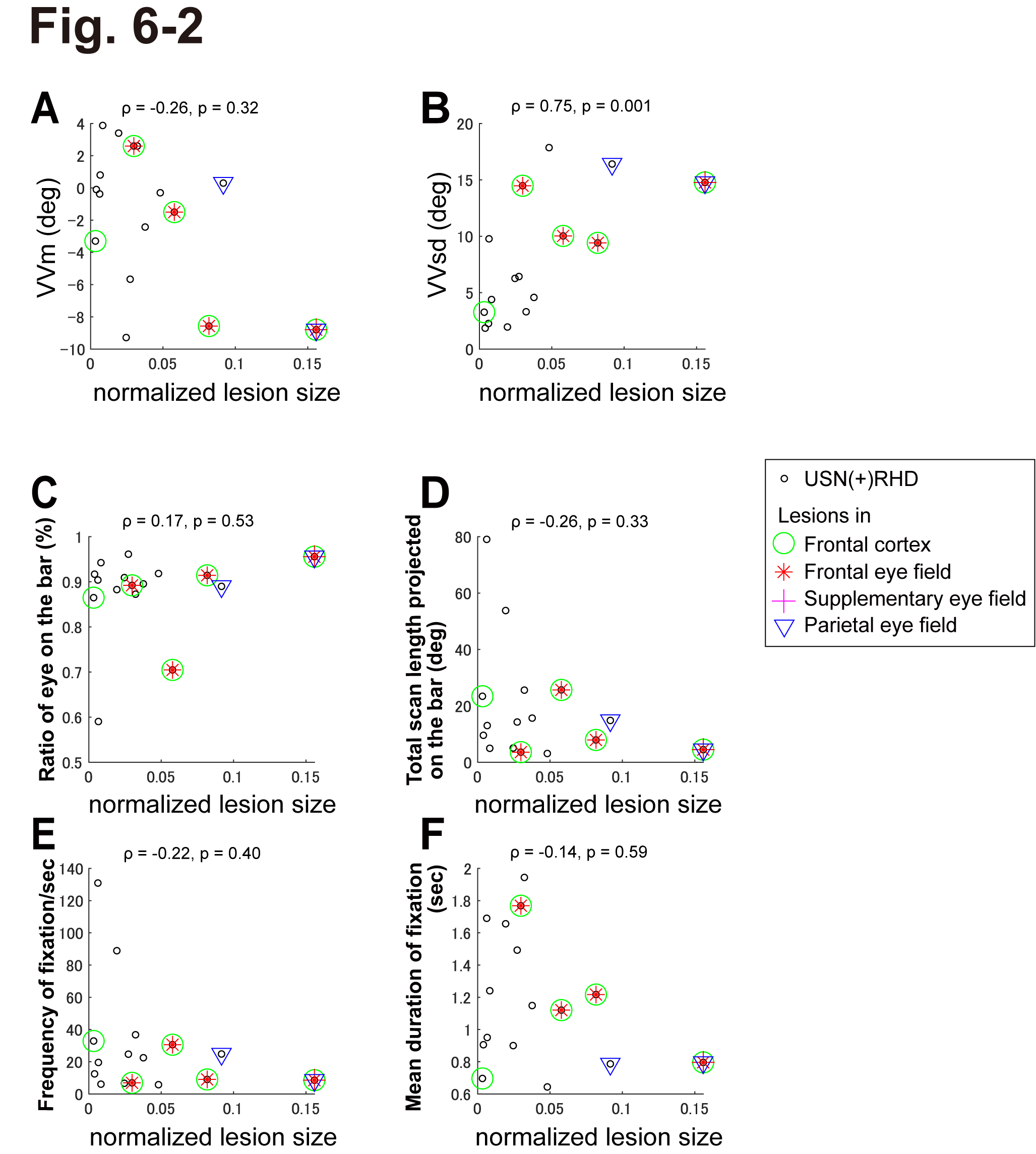

Supplement: Figure 6-2 — Correlation between A. VVm, B. VVsd, C-F. Eye movement parameters and the normalized brain lesion size in USN(+)RHD participants. The format follows that of Fig. 1-1, using the same symbols to represent lesions in specific brain regions for consistency. Download Figure 6-2, TIF file. [file eneuro-12-ENEURO.0279-24.2024-s004.tif]
