# Supplementary material for: Eye Movements during Measurements of Visual Vertical in the Poststroke Subacute Phase
Source: eNeuro. 2025 Jan 16;12(1):ENEURO.0279-24.2024. doi: 10.1523/ENEURO.0279-24.2024 (PMC11747974; doi:10.1523/ENEURO.0279-24.2024)
Supplement: Supplementary file 3 — Statistical Table. Download Statistical Table, DOCX file. [file eneuro-12-ENEURO.0279-24.2024-s005.docx]

| Statistical Table |  |  |  |  |  |
| --- | --- | --- | --- | --- | --- |
| Note that, in the Dunn test, 'no comparison made ' indicates Q<= critical value and they hypothesis is accepted. | | | |  |  |
|  |  |  |  |  |  |
| Figure, Graph | Comparison | Data Structure | Type of test | Power | p-values |
| Fig.1C |  | Non normal distribution | Kruskal-Wallis test | χ2 = 1.936, df = 3, epsilon squared = 0.028 | p = 0.585 |
| Fig.1C | USN(+)RHD vs USN(-)RHD | Non normal distribution | Wilcoxon signed-rank test : Bonferroni’s post hoc test | -0.651 to 0.238 | p = 0.29 |
| Fig.1C | USN(+)RHD vs USN(-)LHD | Non normal distribution | Wilcoxon signed-rank test : Bonferroni’s post hoc test | -0.494 to 0.296 | p = 0.59 |
| Fig.1C | USN(+)RHD vs NC | Non normal distribution | Wilcoxon signed-rank test : Bonferroni’s post hoc test | -0.561 to 0.162 | p = 0.20 |
| Fig.1C | USN(-)RHD vs USN(-)LHD | Non normal distribution | Wilcoxon signed-rank test : Bonferroni’s post hoc test | -0.391 to 0.540 | p = 0.73 |
| Fig.1C | USN(-)RHD vs NC | Non normal distribution | Wilcoxon signed-rank test : Bonferroni’s post hoc test | -0.489 to 0.507 | p = 0.97 |
| Fig.1C | USN(-)LHD vs NC | Non normal distribution | Wilcoxon signed-rank test : Bonferroni’s post hoc test | -0.477 to 0.300 | p = 0.59 |
| Fig.1C | USN(+)RHD | Non normal distribution | One-sample Wilcoxon signed-rank test |  | p = 0.107 |
| Fig.1C | USN(-)RHD | Non normal distribution | One-sample Wilcoxon signed-rank test |  | p = 0.820 |
| Fig.1C | USN(-)LHD | Non normal distribution | One-sample Wilcoxon signed-rank test |  | p = 0.491 |
| Fig.1C | NC | Non normal distribution | One-sample Wilcoxon signed-rank test |  | p = 0.952 |
| Fig.1D |  | Non normal distribution | Kruskal-Wallis test | χ2 = 22.006, df = 3, epsilon squared = 0.328 | p < 0.001 |
| Fig.1D | USN(+)RHD vs USN(-)RHD | Non normal distribution | Wilcoxon signed-rank test : Bonferroni’s post hoc test | 0.245 to 0.870 | p < 0.001 |
| Fig.1D | USN(+)RHD vs USN(-)LHD | Non normal distribution | Wilcoxon signed-rank test : Bonferroni’s post hoc test | 0.548 to 0.941 | p < 0.001 |
| Fig.1D | USN(+)RHD vs NC | Non normal distribution | Wilcoxon signed-rank test : Bonferroni’s post hoc test | 0.290 to 0.866 | p = 0.007 |
| Fig.1D | USN(-)RHD vs USN(-)LHD | Non normal distribution | Wilcoxon signed-rank test : Bonferroni’s post hoc test | -0.133 to 0.742 | p = 0.129 |
| Fig.1D | USN(-)RHD vs NC | Non normal distribution | Wilcoxon signed-rank test : Bonferroni’s post hoc test | -0.440 to 0.426 | p = 0.986 |
| Fig.1D | USN(-)LHD vs NC | Non normal distribution | Wilcoxon signed-rank test : Bonferroni’s post hoc test | -0.652 to 0.032 | p = 0.064 |
| Fig.1D | USN(+)RHD | Non normal distribution | One-sample Wilcoxon signed-rank test |  | p < 0.001 |
| Fig.1D | USN(-)RHD | Non normal distribution | One-sample Wilcoxon signed-rank test |  | p = 0.004 |
| Fig.1D | USN(-)LHD | Non normal distribution | One-sample Wilcoxon signed-rank test |  | p < 0.001 |
| Fig.1D | NC | Non normal distribution | One-sample Wilcoxon signed-rank test |  | p < 0.001 |
| Fig.2B | Group | Non normal distribution | Generalized linear mixed model | χ2 = 8.491, df = 3 | p =0.037 |
| Fig.2B | Trial | Non normal distribution | Generalized linear mixed model | χ2 = 2.168, df = 1 | p = 0.141 |
| Fig.2B | Initial direction | Non normal distribution | Generalized linear mixed model | χ2 = 0.006, df = 1 | p = 0.938 |
| Fig.2B | Group and Initial direction | Non normal distribution | Generalized linear mixed model | χ2 = 2.689, df = 3 | p = 0.442 |
| Fig.2B | USN(+)RHD vs USN(-)RHD | Non normal distribution | Tukey’s method : post hoc test | -0.198 to 0.072 | p = 0.622 |
| Fig.2B | USN(+)RHD vs USN(-)LHD | Non normal distribution | Tukey’s method : post hoc test | -0.099 to 0.139 | p = 0.973 |
| Fig.2B | USN(+)RHD vs NC | Non normal distribution | Tukey’s method : post hoc test | -0.022 to 0.180 | p = 0.184 |
| Fig.2B | USN(-)RHD vs USN(-)LHD | Non normal distribution | Tukey’s method : post hoc test | -0.057 to 0.223 | p = 0.420 |
| Fig.2B | USN(-)RHD vs NC | Non normal distribution | Tukey’s method : post hoc test | -0.110 to 0.141 | p = 0.989 |
| Fig.2B | USN(-)LHD vs NC | Non normal distribution | Tukey’s method : post hoc test | -0.009 to 0.207 | p = 0.085 |
| Fig.2C | Group | Non normal distribution | Generalized linear mixed model | χ2 = 11.087, df = 3 | p =0.011 |
| Fig.2C | Trial | Non normal distribution | Generalized linear mixed model | χ2 = 3.681, df = 1 | p = 0.055 |
| Fig.2C | Initial direction | Non normal distribution | Generalized linear mixed model | χ2 = 109.123, df = 1 | p < 0.001 |
| Fig.2C | Group and Initial direction | Non normal distribution | Generalized linear mixed model | χ2 = 14.216, df = 3 | p = 0.003 |
| Fig.2C | USN(+)RHD vs USN(-)RHD | Non normal distribution | Tukey’s method : post hoc test | -1.343 to 0.520 | p = 0.668 |
| Fig.2C | USN(+)RHD vs USN(-)LHD | Non normal distribution | Tukey’s method : post hoc test | -1.658 to -0.023 | p = 0.041 |
| Fig.2C | USN(+)RHD vs NC | Non normal distribution | Tukey’s method : post hoc test | 0.022 to 1.416 | p = 0.040 |
| Fig.2C | USN(-)RHD vs USN(-)LHD | Non normal distribution | Tukey’s method : post hoc test | -1.397 to 0.539 | p = 0.665 |
| Fig.2C | USN(-)RHD vs NC | Non normal distribution | Tukey’s method : post hoc test | -0.561 to 1.175 | p = 0.800 |
| Fig.2C | USN(-)LHD vs NC | Non normal distribution | Tukey’s method : post hoc test | -0.867 to 0.623 | p = 0.975 |
| Fig.2D | Group | Non normal distribution | Generalized linear mixed model | χ2 = 2.293, df = 3 | p = 0.514 |
| Fig.2D | Trial | Non normal distribution | Generalized linear mixed model | χ2 = 0.084, df = 1 | p = 0.773 |
| Fig.2D | Initial direction | Non normal distribution | Generalized linear mixed model | χ2 = 4.866, df = 1 | p = 0.027 |
| Fig.2D | Group and Initial direction | Non normal distribution | Generalized linear mixed model | χ2 = 3.550, df = 3 | p = 0.314 |
| Fig.2D | USN(+)RHD vs USN(-)RHD | Non normal distribution | Tukey’s method : post hoc test | -0.268 to 0.553 | p = 0.809 |
| Fig.2D | USN(+)RHD vs USN(-)LHD | Non normal distribution | Tukey’s method : post hoc test | -0.448 to 0.276 | p = 0.930 |
| Fig.2D | USN(+)RHD vs NC | Non normal distribution | Tukey’s method : post hoc test | -0.324 to 0.295 | p = 0.999 |
| Fig.2D | USN(-)RHD vs USN(-)LHD | Non normal distribution | Tukey’s method : post hoc test | -0.655 to 0.199 | p = 0.516 |
| Fig.2D | USN(-)RHD vs NC | Non normal distribution | Tukey’s method : post hoc test | -0.255 to 0.512 | p = 0.826 |
| Fig.2D | USN(-)LHD vs NC | Non normal distribution | Tukey’s method : post hoc test | -0.431 to 0.231 | p = 0.865 |
| Fig.2E | Group | Non normal distribution | Generalized linear mixed model | χ2 = 1.375, df = 3 | p = 0.711 |
| Fig.2E | Trial | Non normal distribution | Generalized linear mixed model | χ2 = 0.840, df = 3 | p = 0.359 |
| Fig.2E | Initial direction | Non normal distribution | Generalized linear mixed model | χ2 = 5.157, df = 1 | p = 0.023 |
| Fig.2E | Group and Initial direction | Non normal distribution | Generalized linear mixed model | χ2 = 7.332, df = 3 | p = 0.062 |
| Fig.2E | USN(+)RHD vs USN(-)RHD | Non normal distribution | Tukey’s method : post hoc test | -0.587 to 0.457 | p = 0.989 |
| Fig.2E | USN(+)RHD vs USN(-)LHD | Non normal distribution | Tukey’s method : post hoc test | -0.311 to 0.608 | p = 0.841 |
| Fig.2E | USN(+)RHD vs NC | Non normal distribution | Tukey’s method : post hoc test | -0.420 to 0.366 | p = 0.998 |
| Fig.2E | USN(-)RHD vs USN(-)LHD | Non normal distribution | Tukey’s method : post hoc test | -0.330 to 0.756 | p = 0.744 |
| Fig.2E | USN(-)RHD vs NC | Non normal distribution | Tukey’s method : post hoc test | -0.580 to 0.396 | p = 0.963 |
| Fig.2E | USN(-)LHD vs NC | Non normal distribution | Tukey’s method : post hoc test | -0.299 to 0.542 | p = 0.880 |
| Fig.3Ab Left-tilt,early | | Non normal distribution | Kruskal-Wallis test | χ2 = 80.62, df = 4 | p < 0.001 |
| Fig.3Ab Left-tilt,early | left-down vs left-up | Non normal distribution | Dunn test | Q = 6.92 | p < 0.05 |
| Fig.3Ab Left-tilt,early | left-down vs central | Non normal distribution | Dunn test | Q = 5.86 | p < 0.05 |
| Fig.3Ab Left-tilt,early | left-down vs right-up | Non normal distribution | Dunn test | Q = 0.19 | p > 0.05 |
| Fig.3Ab Left-tilt,early | left-down vs right-down | Non normal distribution | Dunn test | Q = 3.68 | p < 0.05 |
| Fig.3Ab Left-tilt,early | left-up vs central | Non normal distribution | Dunn test | Q = 1.05 | p > 0.05 |
| Fig.3Ab Left-tilt,early | left-up vs right-up | Non normal distribution | Dunn test | Q = 6.72 | p < 0.05 |
| Fig.3Ab Left-tilt,early | left-up vs right-down | Non normal distribution | Dunn test | Q = 3.23 | p < 0.05 |
| Fig.3Ab Left-tilt,early | central vs right-up | Non normal distribution | Dunn test | Q = 5.67 | p < 0.05 |
| Fig.3Ab Left-tilt,early | central vs right-down | Non normal distribution | Dunn test | Q = 2.18 | p > 0.05 |
| Fig.3Ab Left-tilt,early | right-up vs right-down | Non normal distribution | Dunn test | Q = 3.49 | p < 0.05 |
| Fig.3Ab Left-tilt,late | | Non normal distribution | Kruskal-Wallis test | χ2 = 58.20, df = 4 | p < 0.001 |
| Fig.3Ab Left-tilt,late | left-down vs left-up | Non normal distribution | Dunn test | Q = 5.29 | p < 0.05 |
| Fig.3Ab Left-tilt,late | left-down vs central | Non normal distribution | Dunn test | Q = 6.69 | p < 0.05 |
| Fig.3Ab Left-tilt,late | left-down vs right-up | Non normal distribution | Dunn test | Q = 1.69 | p > 0.05 |
| Fig.3Ab Left-tilt,late | left-down vs right-down | Non normal distribution | Dunn test | Q = 2.91 | p < 0.05 |
| Fig.3Ab Left-tilt,late | left-up vs central | Non normal distribution | Dunn test | Q = 1.39 | p > 0.05 |
| Fig.3Ab Left-tilt,late | left-up vs right-up | Non normal distribution | Dunn test | Q = 3.61 | p < 0.05 |
| Fig.3Ab Left-tilt,late | left-up vs right-down | Non normal distribution | Dunn test | Q = 2.39 | p > 0.05 |
| Fig.3Ab Left-tilt,late | central vs right-up | Non normal distribution | Dunn test | Q = 5.00 | p < 0.05 |
| Fig.3Ab Left-tilt,late | central vs right-down | Non normal distribution | Dunn test | Q = 3.78 | p < 0.05 |
| Fig.3Ab Left-tilt,late | right-up vs right-down | Non normal distribution | Dunn test | Q = 1.22 | p > 0.05 |
| Fig.3Ad Right-tilt,early | | Non normal distribution | Kruskal-Wallis test | χ2 = 77.81, df = 4 | p < 0.001 |
| Fig.3Ad Right-tilt,early | left-down vs left-up | Non normal distribution | Dunn test | Q = 1.45 | p > 0.05 |
| Fig.3Ad Right-tilt,early | left-down vs central | Non normal distribution | Dunn test | Q = 3.53 | p < 0.05 |
| Fig.3Ad Right-tilt,early | left-down vs right-up | Non normal distribution | Dunn test | Q = 5.62 | p < 0.05 |
| Fig.3Ad Right-tilt,early | left-down vs right-down | Non normal distribution | Dunn test | no comparison made |  |
| Fig.3Ad Right-tilt,early | left-up vs central | Non normal distribution | Dunn test | Q = 4.98 | p < 0.05 |
| Fig.3Ad Right-tilt,early | left-up vs right-up | Non normal distribution | Dunn test | Q = 7.08 | p < 0.05 |
| Fig.3Ad Right-tilt,early | left-up vs right-down | Non normal distribution | Dunn test | Q = 0.31 | p > 0.05 |
| Fig.3Ad Right-tilt,early | central vs right-up | Non normal distribution | Dunn test | Q = 2.10 | p > 0.05 |
| Fig.3Ad Right-tilt,early | central vs right-down | Non normal distribution | Dunn test | Q = 4.67 | p < 0.05 |
| Fig.3Ad Right-tilt,early | right-up vs right-down | Non normal distribution | Dunn test | Q = 6.67 | p < 0.05 |
| Fig.3Ad Right-tilt,late | | Non normal distribution | Kruskal-Wallis test | χ2 = 51.60, df = 4 | p < 0.001 |
| Fig.3Ad Right-tilt,late | left-down vs left-up | Non normal distribution | Dunn test | Q = 1.45 | p > 0.05 |
| Fig.3Ad Right-tilt,late | left-down vs central | Non normal distribution | Dunn test | Q = 5.57 | p < 0.05 |
| Fig.3Ad Right-tilt,late | left-down vs right-up | Non normal distribution | Dunn test | Q = 4.96 | p < 0.05 |
| Fig.3Ad Right-tilt,late | left-down vs right-down | Non normal distribution | Dunn test | Q = 0.76 | p > 0.05 |
| Fig.3Ad Right-tilt,late | left-up vs central | Non normal distribution | Dunn test | Q = 4.12 | p < 0.05 |
| Fig.3Ad Right-tilt,late | left-up vs right-up | Non normal distribution | Dunn test | Q = 3.51 | p < 0.05 |
| Fig.3Ad Right-tilt,late | left-up vs right-down | Non normal distribution | Dunn test | no comparison made |  |
| Fig.3Ad Right-tilt,late | central vs right-up | Non normal distribution | Dunn test | Q = 0.61 | p > 0.05 |
| Fig.3Ad Right-tilt,late | central vs right-down | Non normal distribution | Dunn test | Q = 4.80 | p < 0.05 |
| Fig.3Ad Right-tilt,late | right-up vs right-down | Non normal distribution | Dunn test | Q = 4.19 | p < 0.05 |
| Fig.4Ab Left-tilt,early | | Non normal distribution | Kruskal-Wallis test | χ2 = 29.92, df = 4 | p < 0.001 |
| Fig.4Ab Left-tilt,early | left-down vs left-up | Non normal distribution | Dunn test | Q = 4.28 | p < 0.05 |
| Fig.4Ab Left-tilt,early | left-down vs central | Non normal distribution | Dunn test | Q = 3.04 | p < 0.05 |
| Fig.4Ab Left-tilt,early | left-down vs right-up | Non normal distribution | Dunn test | Q = 0.09 | p > 0.05 |
| Fig.4Ab Left-tilt,early | left-down vs right-down | Non normal distribution | Dunn test | Q = 3.05 | p < 0.05 |
| Fig.4Ab Left-tilt,early | left-up vs central | Non normal distribution | Dunn test | Q = 1.24 | p > 0.05 |
| Fig.4Ab Left-tilt,early | left-up vs right-up | Non normal distribution | Dunn test | Q = 4.18 | p < 0.05 |
| Fig.4Ab Left-tilt,early | left-up vs right-down | Non normal distribution | Dunn test | no comparison made |  |
| Fig.4Ab Left-tilt,early | central vs right-up | Non normal distribution | Dunn test | Q = 2.94 | p < 0.05 |
| Fig.4Ab Left-tilt,early | central vs right-down | Non normal distribution | Dunn test | Q = 0.01 | p > 0.05 |
| Fig.4Ab Left-tilt,early | right-up vs right-down | Non normal distribution | Dunn test | Q = 2.96 | p < 0.05 |
| Fig.4Ab Left-tilt,late | | Non normal distribution | Kruskal-Wallis test | χ2 = 20.30, df = 4 | p < 0.001 |
| Fig.4Ab Left-tilt,late | left-down vs left-up | Non normal distribution | Dunn test | Q = 3.95 | p < 0.05 |
| Fig.4Ab Left-tilt,late | left-down vs central | Non normal distribution | Dunn test | Q = 1.38 | p > 0.05 |
| Fig.4Ab Left-tilt,late | left-down vs right-up | Non normal distribution | Dunn test | Q = 0.10 | p > 0.05 |
| Fig.4Ab Left-tilt,late | left-down vs right-down | Non normal distribution | Dunn test | Q = 1.34 | p > 0.05 |
| Fig.4Ab Left-tilt,late | left-up vs central | Non normal distribution | Dunn test | no comparison made |  |
| Fig.4Ab Left-tilt,late | left-up vs right-up | Non normal distribution | Dunn test | Q = 3.85 | p < 0.05 |
| Fig.4Ab Left-tilt,late | left-up vs right-down | Non normal distribution | Dunn test | Q = 2.61 | p > 0.05 |
| Fig.4Ab Left-tilt,late | central vs right-up | Non normal distribution | Dunn test | no comparison made |  |
| Fig.4Ab Left-tilt,late | central vs right-down | Non normal distribution | Dunn test | no comparison made |  |
| Fig.4Ab Left-tilt,late | right-up vs right-down | Non normal distribution | Dunn test | no comparison made |  |
| Fig.4Ad Right-tilt,early | | Non normal distribution | Kruskal-Wallis test | χ2 = 38.85, df = 4 | p < 0.001 |
| Fig.4Ad Right-tilt,early | left-down vs left-up | Non normal distribution | Dunn test | no comparison made |  |
| Fig.4Ad Right-tilt,early | left-down vs central | Non normal distribution | Dunn test | no comparison made |  |
| Fig.4Ad Right-tilt,early | left-down vs right-up | Non normal distribution | Dunn test | Q = 4.58 | p < 0.05 |
| Fig.4Ad Right-tilt,early | left-down vs right-down | Non normal distribution | Dunn test | Q = 1.11 | p > 0.05 |
| Fig.4Ad Right-tilt,early | left-up vs central | Non normal distribution | Dunn test | no comparison made |  |
| Fig.4Ad Right-tilt,early | left-up vs right-up | Non normal distribution | Dunn test | Q = 4.70 | p < 0.05 |
| Fig.4Ad Right-tilt,early | left-up vs right-down | Non normal distribution | Dunn test | Q = 1.00 | p > 0.05 |
| Fig.4Ad Right-tilt,early | central vs right-up | Non normal distribution | Dunn test | Q = 3.64 | p < 0.05 |
| Fig.4Ad Right-tilt,early | central vs right-down | Non normal distribution | Dunn test | Q = 2.05 | p > 0.05 |
| Fig.4Ad Right-tilt,early | right-up vs right-down | Non normal distribution | Dunn test | Q = 5.69 | p < 0.05 |
| Fig.4Ad Right-tilt,late | | Non normal distribution | Kruskal-Wallis test | χ2 = 23.77, df = 4 | p < 0.001 |
| Fig.4Ad Right-tilt,late | left-down vs left-up | Non normal distribution | Dunn test | no comparison made |  |
| Fig.4Ad Right-tilt,late | left-down vs central | Non normal distribution | Dunn test | no comparison made |  |
| Fig.4Ad Right-tilt,late | left-down vs right-up | Non normal distribution | Dunn test | Q = 4.13 | p < 0.05 |
| Fig.4Ad Right-tilt,late | left-down vs right-down | Non normal distribution | Dunn test | Q = 0.08 | p > 0.05 |
| Fig.4Ad Right-tilt,late | left-up vs central | Non normal distribution | Dunn test | no comparison made |  |
| Fig.4Ad Right-tilt,late | left-up vs right-up | Non normal distribution | Dunn test | Q = 2.68 | p > 0.05 |
| Fig.4Ad Right-tilt,late | left-up vs right-down | Non normal distribution | Dunn test | Q = 1.52 | p > 0.05 |
| Fig.4Ad Right-tilt,late | central vs right-up | Non normal distribution | Dunn test | Q = 3.38 | p < 0.05 |
| Fig.4Ad Right-tilt,late | central vs right-down | Non normal distribution | Dunn test | Q = 0.82 | p > 0.05 |
| Fig.4Ad Right-tilt,late | right-up vs right-down | Non normal distribution | Dunn test | Q = 4.20 | p < 0.05 |
| Fig.4Bb Left-tilt,early | | Non normal distribution | Kruskal-Wallis test | χ2 = 28.04, df = 4 | p < 0.001 |
| Fig.4Bb Left-tilt,early | left-down vs left-up | Non normal distribution | Dunn test | Q = 3.57 | p < 0.05 |
| Fig.4Bb Left-tilt,early | left-down vs central | Non normal distribution | Dunn test | Q = 4.52 | p < 0.05 |
| Fig.4Bb Left-tilt,early | left-down vs right-up | Non normal distribution | Dunn test | Q = 0.88 | p > 0.05 |
| Fig.4Bb Left-tilt,early | left-down vs right-down | Non normal distribution | Dunn test | Q = 2.73 | p > 0.05 |
| Fig.4Bb Left-tilt,early | left-up vs central | Non normal distribution | Dunn test | no comparison made |  |
| Fig.4Bb Left-tilt,early | left-up vs right-up | Non normal distribution | Dunn test | Q = 2.68 | p > 0.05 |
| Fig.4Bb Left-tilt,early | left-up vs right-down | Non normal distribution | Dunn test | no comparison made |  |
| Fig.4Bb Left-tilt,early | central vs right-up | Non normal distribution | Dunn test | Q = 3.64 | p < 0.05 |
| Fig.4Bb Left-tilt,early | central vs right-down | Non normal distribution | Dunn test | Q = 1.79 | p > 0.05 |
| Fig.4Bb Left-tilt,early | right-up vs right-down | Non normal distribution | Dunn test | no comparison made |  |
| Fig.4Bb Left-tilt,late | | Non normal distribution | Kruskal-Wallis test | χ2 = 23.74, df = 4 | p < 0.001 |
| Fig.4Bb Left-tilt,late | left-down vs left-up | Non normal distribution | Dunn test | Q = 2.00 | p > 0.05 |
| Fig.4Bb Left-tilt,late | left-down vs central | Non normal distribution | Dunn test | Q = 4.32 | p < 0.05 |
| Fig.4Bb Left-tilt,late | left-down vs right-up | Non normal distribution | Dunn test | Q = 0.69 | p > 0.05 |
| Fig.4Bb Left-tilt,late | left-down vs right-down | Non normal distribution | Dunn test | Q = 0.65 | p > 0.05 |
| Fig.4Bb Left-tilt,late | left-up vs central | Non normal distribution | Dunn test | Q = 2.33 | p > 0.05 |
| Fig.4Bb Left-tilt,late | left-up vs right-up | Non normal distribution | Dunn test | no comparison made |  |
| Fig.4Bb Left-tilt,late | left-up vs right-down | Non normal distribution | Dunn test | no comparison made |  |
| Fig.4Bb Left-tilt,late | central vs right-up | Non normal distribution | Dunn test | Q = 3.64 | p < 0.05 |
| Fig.4Bb Left-tilt,late | central vs right-down | Non normal distribution | Dunn test | Q = 3.68 | p < 0.05 |
| Fig.4Bb Left-tilt,late | right-up vs right-down | Non normal distribution | Dunn test | no comparison made |  |
| Fig.4Bd Right-tilt,early | | Non normal distribution | Kruskal-Wallis test | χ2 = 30.82, df = 4 | p < 0.001 |
| Fig.4Bd Right-tilt,early | left-down vs left-up | Non normal distribution | Dunn test | no comparison made |  |
| Fig.4Bd Right-tilt,early | left-down vs central | Non normal distribution | Dunn test | Q = 1.98 | p > 0.05 |
| Fig.4Bd Right-tilt,early | left-down vs right-up | Non normal distribution | Dunn test | Q = 1.06 | p > 0.05 |
| Fig.4Bd Right-tilt,early | left-down vs right-down | Non normal distribution | Dunn test | Q = 2.47 | p > 0.05 |
| Fig.4Bd Right-tilt,early | left-up vs central | Non normal distribution | Dunn test | Q = 4.16 | p < 0.05 |
| Fig.4Bd Right-tilt,early | left-up vs right-up | Non normal distribution | Dunn test | Q = 3.25 | p < 0.05 |
| Fig.4Bd Right-tilt,early | left-up vs right-down | Non normal distribution | Dunn test | Q = 0.28 | p > 0.05 |
| Fig.4Bd Right-tilt,early | central vs right-up | Non normal distribution | Dunn test | no comparison made |  |
| Fig.4Bd Right-tilt,early | central vs right-down | Non normal distribution | Dunn test | Q = 4.45 | p < 0.05 |
| Fig.4Bd Right-tilt,early | right-up vs right-down | Non normal distribution | Dunn test | Q = 3.53 | p < 0.05 |
| Fig.4Bd Right-tilt,late | | Non normal distribution | Kruskal-Wallis test | χ2 = 23,74, df = 4 | p < 0.001 |
| Fig.4Bd Right-tilt,late | left-down vs left-up | Non normal distribution | Dunn test | no comparison made |  |
| Fig.4Bd Right-tilt,late | left-down vs central | Non normal distribution | Dunn test | Q = 3.16 | p < 0.05 |
| Fig.4Bd Right-tilt,late | left-down vs right-up | Non normal distribution | Dunn test | no comparison made |  |
| Fig.4Bd Right-tilt,late | left-down vs right-down | Non normal distribution | Dunn test | Q = 1.54 | p > 0.05 |
| Fig.4Bd Right-tilt,late | left-up vs central | Non normal distribution | Dunn test | Q = 3.31 | p < 0.05 |
| Fig.4Bd Right-tilt,late | left-up vs right-up | Non normal distribution | Dunn test | no comparison made |  |
| Fig.4Bd Right-tilt,late | left-up vs right-down | Non normal distribution | Dunn test | Q = 1.38 | p > 0.05 |
| Fig.4Bd Right-tilt,late | central vs right-up | Non normal distribution | Dunn test | Q = 2.99 | p < 0.05 |
| Fig.4Bd Right-tilt,late | central vs right-down | Non normal distribution | Dunn test | Q = 4.70 | p < 0.05 |
| Fig.4Bd Right-tilt,late | right-up vs right-down | Non normal distribution | Dunn test | Q = 1.71 | p > 0.05 |
| Fig.4Cb Left-tilt,early | | Non normal distribution | Kruskal-Wallis test | χ2 = 27.78, df = 4 | p < 0.001 |
| Fig.4Cb Left-tilt,early | left-down vs left-up | Non normal distribution | Dunn test | Q = 3.02 | p < 0.05 |
| Fig.4Cb Left-tilt,early | left-down vs central | Non normal distribution | Dunn test | Q = 4.46 | p < 0.05 |
| Fig.4Cb Left-tilt,early | left-down vs right-up | Non normal distribution | Dunn test | Q = 0.41 | p > 0.05 |
| Fig.4Cb Left-tilt,early | left-down vs right-down | Non normal distribution | Dunn test | Q = 2.56 | p > 0.05 |
| Fig.4Cb Left-tilt,early | left-up vs central | Non normal distribution | Dunn test | no comparison made |  |
| Fig.4Cb Left-tilt,early | left-up vs right-up | Non normal distribution | Dunn test | Q = 2.61 | p > 0.05 |
| Fig.4Cb Left-tilt,early | left-up vs right-down | Non normal distribution | Dunn test | no comparison made |  |
| Fig.4Cb Left-tilt,early | central vs right-up | Non normal distribution | Dunn test | Q = 4.05 | p < 0.05 |
| Fig.4Cb Left-tilt,early | central vs right-down | Non normal distribution | Dunn test | Q = 1.90 | p > 0.05 |
| Fig.4Cb Left-tilt,early | right-up vs right-down | Non normal distribution | Dunn test | no comparison made |  |
| Fig.4Cb Left-tilt,late | | Non normal distribution | Kruskal-Wallis test | χ2 = 21.82, df = 4 | p < 0.001 |
| Fig.4Cb Left-tilt,late | left-down vs left-up | Non normal distribution | Dunn test | Q = 2.32 | p > 0.05 |
| Fig.4Cb Left-tilt,late | left-down vs central | Non normal distribution | Dunn test | Q = 4.06 | p < 0.05 |
| Fig.4Cb Left-tilt,late | left-down vs right-up | Non normal distribution | Dunn test | Q = 0.40 | p > 0.05 |
| Fig.4Cb Left-tilt,late | left-down vs right-down | Non normal distribution | Dunn test | Q = 2.39 | p > 0.05 |
| Fig.4Cb Left-tilt,late | left-up vs central | Non normal distribution | Dunn test | Q = 1.74 | p > 0.05 |
| Fig.4Cb Left-tilt,late | left-up vs right-up | Non normal distribution | Dunn test | no comparison made |  |
| Fig.4Cb Left-tilt,late | left-up vs right-down | Non normal distribution | Dunn test | no comparison made |  |
| Fig.4Cb Left-tilt,late | central vs right-up | Non normal distribution | Dunn test | Q = 3.66 | p < 0.05 |
| Fig.4Cb Left-tilt,late | central vs right-down | Non normal distribution | Dunn test | no comparison made |  |
| Fig.4Cb Left-tilt,late | right-up vs right-down | Non normal distribution | Dunn test | no comparison made |  |
| Fig.4Cd Right-tilt,early | | Non normal distribution | Kruskal-Wallis test | χ2 = 25.91, df = 4 | p < 0.001 |
| Fig.4Cd Right-tilt,early | left-down vs left-up | Non normal distribution | Dunn test | Q = 1.92 | p > 0.05 |
| Fig.4Cd Right-tilt,early | left-down vs central | Non normal distribution | Dunn test | no comparison made |  |
| Fig.4Cd Right-tilt,early | left-down vs right-up | Non normal distribution | Dunn test | no comparison made |  |
| Fig.4Cd Right-tilt,early | left-down vs right-down | Non normal distribution | Dunn test | no comparison made |  |
| Fig.4Cd Right-tilt,early | left-up vs central | Non normal distribution | Dunn test | Q = 4.45 | p < 0.05 |
| Fig.4Cd Right-tilt,early | left-up vs right-up | Non normal distribution | Dunn test | Q = 3.94 | p < 0.05 |
| Fig.4Cd Right-tilt,early | left-up vs right-down | Non normal distribution | Dunn test | Q = 1.80 | p > 0.05 |
| Fig.4Cd Right-tilt,early | central vs right-up | Non normal distribution | Dunn test | no comparison made |  |
| Fig.4Cd Right-tilt,early | central vs right-down | Non normal distribution | Dunn test | Q = 2.65 | p > 0.05 |
| Fig.4Cd Right-tilt,early | right-up vs right-down | Non normal distribution | Dunn test | Q = 2.15 | p > 0.05 |
| Fig.4Cd Right-tilt,late | | Non normal distribution | Kruskal-Wallis test | χ2 = 24.95, df = 4 | p < 0.001 |
| Fig.4Cd Right-tilt,late | left-down vs left-up | Non normal distribution | Dunn test | Q = 0.38 | p > 0.05 |
| Fig.4Cd Right-tilt,late | left-down vs central | Non normal distribution | Dunn test | Q = 3.97 | p < 0.05 |
| Fig.4Cd Right-tilt,late | left-down vs right-up | Non normal distribution | Dunn test | no comparison made |  |
| Fig.4Cd Right-tilt,late | left-down vs right-down | Non normal distribution | Dunn test | no comparison made |  |
| Fig.4Cd Right-tilt,late | left-up vs central | Non normal distribution | Dunn test | Q = 4.34 | p < 0.05 |
| Fig.4Cd Right-tilt,late | left-up vs right-up | Non normal distribution | Dunn test | Q = 2.12 | p > 0.05 |
| Fig.4Cd Right-tilt,late | left-up vs right-down | Non normal distribution | Dunn test | Q = 0.79 | p > 0.05 |
| Fig.4Cd Right-tilt,late | central vs right-up | Non normal distribution | Dunn test | Q = 2.23 | p > 0.05 |
| Fig.4Cd Right-tilt,late | central vs right-down | Non normal distribution | Dunn test | Q = 3.55 | p < 0.05 |
| Fig.4Cd Right-tilt,late | right-up vs right-down | Non normal distribution | Dunn test | No comparision |  |
| Fig.6D, 6E | FEF lesion and VV improvement | Non normal distribution | Fisher's exact test |  | p = 0.005 |
| Fig.6D, 6E | SEF lesion and VV improvement | Non normal distribution | Fisher's exact test |  | p = 0.40 |
| Fig.6D, 6E | PEF lesion and VV improvement | Non normal distribution | Fisher's exact test |  | p = 1.00 |
| Fig.6D, 6E | difference in lesion size between groups | Non normal distribution | Wilcoxon rank-sum test |  | p = 0.171 |
| Fig.7Ab Left-tilt,early | | Non normal distribution | Kruskal-Wallis test | χ2 = 4.78, df = 4 | p = 0.310 |
| Fig.7Ab Left-tilt,late | | Non normal distribution | Kruskal-Wallis test | χ2 = 9.73, df = 4 | p = 0.045 |
| Fig.7Ab Left-tilt,late | left-down vs left-up | Non normal distribution | Dunn test | no comparison made |  |
| Fig.7Ab Left-tilt,late | left-down vs central | Non normal distribution | Dunn test | no comparison made |  |
| Fig.7Ab Left-tilt,late | left-down vs right-up | Non normal distribution | Dunn test | Q = 0.37 | p > 0.05 |
| Fig.7Ab Left-tilt,late | left-down vs right-down | Non normal distribution | Dunn test | no comparison made |  |
| Fig.7Ab Left-tilt,late | left-up vs central | Non normal distribution | Dunn test | no comparison made |  |
| Fig.7Ab Left-tilt,late | left-up vs right-up | Non normal distribution | Dunn test | Q = 2.37 | p > 0.05 |
| Fig.7Ab Left-tilt,late | left-up vs right-down | Non normal distribution | Dunn test | no comparison made |  |
| Fig.7Ab Left-tilt,late | central vs right-up | Non normal distribution | Dunn test | Q = 2.20 | p > 0.05 |
| Fig.7Ab Left-tilt,late | central vs right-down | Non normal distribution | Dunn test | no comparison made |  |
| Fig.7Ab Left-tilt,late | right-up vs right-down | Non normal distribution | Dunn test | Q = 0.55 | p > 0.05 |
| Fig.7Ad Right-tilt,early | | Non normal distribution | Kruskal-Wallis test | χ2 = 14.50, df = 4 | p = 0.006 |
| Fig.7Ad Right-tilt,early | left-down vs left-up | Non normal distribution | Dunn test | no comparison made |  |
| Fig.7Ad Right-tilt,early | left-down vs central | Non normal distribution | Dunn test | no comparison made |  |
| Fig.7Ad Right-tilt,early | left-down vs right-up | Non normal distribution | Dunn test | Q = 3.06 | p < 0.05 |
| Fig.7Ad Right-tilt,early | left-down vs right-down | Non normal distribution | Dunn test | Q = 0.43 | p > 0.05 |
| Fig.7Ad Right-tilt,early | left-up vs central | Non normal distribution | Dunn test | no comparison made |  |
| Fig.7Ad Right-tilt,early | left-up vs right-up | Non normal distribution | Dunn test | no comparison made | p > 0.05 |
| Fig.7Ad Right-tilt,early | left-up vs right-down | Non normal distribution | Dunn test | Q = 1.51 | p > 0.05 |
| Fig.7Ad Right-tilt,early | central vs right-up | Non normal distribution | Dunn test | Q = 2.17 | p > 0.05 |
| Fig.7Ad Right-tilt,early | central vs right-down | Non normal distribution | Dunn test | Q = 1.32 | p > 0.05 |
| Fig.7Ad Right-tilt,early | right-up vs right-down | Non normal distribution | Dunn test | Q = 3.48 | p < 0.05 |
| Fig.7Ad Right-tilt,late | | Non normal distribution | Kruskal-Wallis test | χ2 = 9.08, df = 4 | p = 0.059 |
| Fig.7Bb Left-tilt,early | | Non normal distribution | Kruskal-Wallis test | χ2 = 5.76, df = 4 | p = 0.218 |
| Fig.7Bb Left-tilt,late | | Non normal distribution | Kruskal-Wallis test | χ2 = 5.01, df = 4 | p = 0.287 |
| Fig.7Bd Right-tilt,early | | Non normal distribution | Kruskal-Wallis test | χ2 = 19.91, df = 4 | p < 0.001 |
| Fig.7Bd Right-tilt,early | left-down vs left-up | Non normal distribution | Dunn test | Q = 1.47 | p > 0.05 |
| Fig.7Bd Right-tilt,early | left-down vs central | Non normal distribution | Dunn test | Q = 2.02 | p > 0.05 |
| Fig.7Bd Right-tilt,early | left-down vs right-up | Non normal distribution | Dunn test | Q = 3.91 | p < 0.05 |
| Fig.7Bd Right-tilt,early | left-down vs right-down | Non normal distribution | Dunn test | Q = 0.22 | p > 0.05 |
| Fig.7Bd Right-tilt,early | left-up vs central | Non normal distribution | Dunn test | no comparison made |  |
| Fig.7Bd Right-tilt,early | left-up vs right-up | Non normal distribution | Dunn test | Q = 2.43 | p > 0.05 |
| Fig.7Bd Right-tilt,early | left-up vs right-down | Non normal distribution | Dunn test | no comparison made |  |
| Fig.7Bd Right-tilt,early | central vs right-up | Non normal distribution | Dunn test | no comparison made |  |
| Fig.7Bd Right-tilt,early | central vs right-down | Non normal distribution | Dunn test | no comparison made |  |
| Fig.7Bd Right-tilt,early | right-up vs right-down | Non normal distribution | Dunn test | Q = 3.69 | p < 0.05 |
| Fig.7Bd Right-tilt,late | | Non normal distribution | Kruskal-Wallis test | χ2 = 5.78, df = 4 | p = 0.233 |
| Fig.7Cb Left-tilt,early | | Non normal distribution | Kruskal-Wallis test | χ2 = 12.93, df = 4 | p = 0.012 |
| Fig.7Cb Left-tilt,early | left-down vs left-up | Non normal distribution | Dunn test | Q = 3.20 | p < 0.05 |
| Fig.7Cb Left-tilt,early | left-down vs central | Non normal distribution | Dunn test | Q = 1.70 | p > 0.05 |
| Fig.7Cb Left-tilt,early | left-down vs right-up | Non normal distribution | Dunn test | Q = 0.30 | p > 0.05 |
| Fig.7Cb Left-tilt,early | left-down vs right-down | Non normal distribution | Dunn test | Q = 1.23 | p > 0.05 |
| Fig.7Cb Left-tilt,early | left-up vs central | Non normal distribution | Dunn test | no comparison made |  |
| Fig.7Cb Left-tilt,early | left-up vs right-up | Non normal distribution | Dunn test | Q = 2.90 | p < 0.05 |
| Fig.7Cb Left-tilt,early | left-up vs right-down | Non normal distribution | Dunn test | Q = 1.97 | p > 0.05 |
| Fig.7Cb Left-tilt,early | central vs right-up | Non normal distribution | Dunn test | no comparison made |  |
| Fig.7Cb Left-tilt,early | central vs right-down | Non normal distribution | Dunn test | no comparison made |  |
| Fig.7Cb Left-tilt,early | right-up vs right-down | Non normal distribution | Dunn test | no comparison made |  |
| Fig.7Cb Left-tilt,late | | Non normal distribution | Kruskal-Wallis test | χ2 = 10.13, df = 4 | p = 0.038 |
| Fig.7Cb Left-tilt,late | left-down vs left-up | Non normal distribution | Dunn test | no comparison made |  |
| Fig.7Cb Left-tilt,late | left-down vs central | Non normal distribution | Dunn test | no comparison made |  |
| Fig.7Cb Left-tilt,late | left-down vs right-up | Non normal distribution | Dunn test | Q = 0.49 | p > 0.05 |
| Fig.7Cb Left-tilt,late | left-down vs right-down | Non normal distribution | Dunn test | no comparison made |  |
| Fig.7Cb Left-tilt,late | left-up vs central | Non normal distribution | Dunn test | no comparison made |  |
| Fig.7Cb Left-tilt,late | left-up vs right-up | Non normal distribution | Dunn test | Q = 2.84 | p < 0.05 |
| Fig.7Cb Left-tilt,late | left-up vs right-down | Non normal distribution | Dunn test | Q = 2.35 | p > 0.05 |
| Fig.7Cb Left-tilt,late | central vs right-up | Non normal distribution | Dunn test | Q = 0.49 | p > 0.05 |
| Fig.7Cb Left-tilt,late | central vs right-down | Non normal distribution | Dunn test | no comparison made |  |
| Fig.7Cb Left-tilt,late | right-up vs right-down | Non normal distribution | Dunn test | Q = 0.49 | p > 0.05 |
| Fig.7Cd Right-tilt,early | | Non normal distribution | Kruskal-Wallis test | χ2 = 24.95, df = 4 | p = 0.065 |
| Fig.7Cd Right-tilt,late | | Non normal distribution | Kruskal-Wallis test | χ2 = 5.50, df = 4 | p = 0.240 |
| Fig.1-1A Extended data | VVm and Line Bisection test | Non normal distribution | Spearman's rank correlation | ρ = -0.05 | p = 0.86 |
| Fig.1-1A Extended data | VVm and Star cancellation test | Non normal distribution | Spearman's rank correlation | ρ = -0.13 | p = 0.65 |
| Fig.1-1A Extended data | VVm and Flower copying test | Non normal distribution | Spearman's rank correlation | ρ = 0.08 | p = 0.75 |
| Fig.1-1B Extended data | VVsd and Line Bisection test | Non normal distribution | Spearman's rank correlation | ρ = -0.64 | p = 0.007 |
| Fig.1-1B Extended data | VVsd and Star cancellation test | Non normal distribution | Spearman's rank correlation | ρ = -0.54 | p = 0.031 |
| Fig.1-1B Extended data | VVsd and Flower copying test | Non normal distribution | Spearman's rank correlation | ρ = -0.45 | p = 0.078 |
| Fig.2-1-A Extended data | Ratio of eye on the bar and VVm | Non normal distribution | Spearman's rank correlation | ρ = -0.32 | p = 0.23 |
| Fig.21A Extended data | Total scan length projected on the bar and VVm | Non normal distribution | Spearman's rank correlation | ρ = 0.11 | p = 0.68 |
| Fig.2-1A Extended data | Frequency of fixation and VVm | Non normal distribution | Spearman's rank correlation | ρ = 0.12 | p = 0.65 |
| Fig.2-1A Extended data | Mean duration of fixation | Non normal distribution | Spearman's rank correlation | ρ = 0.41 | p = 0.106 |
| Fig.2-1B Extended data | Ratio of eye on the bar and VVsd | Non normal distribution | Spearman's rank correlation | ρ = 0.10 | p = 0.72 |
| Fig.2-1B Extended data | Total scan length projected on the bar and VVsd | Non normal distribution | Spearman's rank correlation | ρ = -0.55 | p = 0.031 |
| Fig.2-1B Extended data | Frequency of fixation and VVsd | Non normal distribution | Spearman's rank correlation | ρ = -0.48 | p = 0.059 |
| Fig.2-1B Extended data | Mean duration of fixation and VVsd | Non normal distribution | Spearman's rank correlation | ρ = -0.39 | p = 0.138 |
| Fig.6-2A Extended data | VVm and normalized lesion size | Non normal distribution | Spearman's rank correlation | ρ = -0.26 | p = 0.32 |
| Fig.6-2B Extended data | VVsd and normalized lesion size | Non normal distribution | Spearman's rank correlation | ρ = 0.75 | p = 0.001 |
| Fig.6-2C Extended data | Ratio of eye on the bar and normalized lesion size | Non normal distribution | Spearman's rank correlation | ρ = 0.17 | p = 0.53 |
| Fig.6-2D Extended data | Total scan length projected on the bar and normalized lesion size | Non normal distribution | Spearman's rank correlation | ρ = -0.26 | p = 0.33 |
| Fig.6-2E Extended data | Frequency of fixation and normalized lesion size | Non normal distribution | Spearman's rank correlation | ρ = -0.22 | p = 0.40 |
| Fig.6-2F Extended data | Mean duration of fixation and normalized lesion size | Non normal distribution | Spearman's rank correlation | ρ = -0.14 | p = 0.59 |
